# Dual Predation by Bacteriophage and *Bdellovibrio bacteriovorus* Can Eradicate *Escherichia coli* Prey in Situations where Single Predation Cannot

**DOI:** 10.1128/JB.00629-19

**Published:** 2020-02-25

**Authors:** Laura Hobley, J. Kimberley Summers, Rob Till, David S. Milner, Robert J. Atterbury, Amy Stroud, Michael J. Capeness, Stephanie Gray, Andreas Leidenroth, Carey Lambert, Ian Connerton, Jamie Twycross, Michelle Baker, Jess Tyson, Jan-Ulrich Kreft, R. Elizabeth Sockett

**Affiliations:** a School of Life Sciences, University of Nottingham, Nottingham, United Kingdom; b Institute of Microbiology and Infection and Centre for Computational Biology and School of Biosciences, University of Birmingham, Birmingham, United Kingdom; c School of Biosciences, University of Nottingham, Loughborough, United Kingdom; d School of Computer Science, University of Nottingham, Nottingham, United Kingdom; Geisel School of Medicine at Dartmouth

**Keywords:** *Bdellovibrio*, bacteriophage, RTP phage, predation, cooperation, predator prey models, mathematical modeling, approximate Bayesian computation, cooperation, predator-prey models

## Abstract

Bacteria are preyed upon by diverse microbial predators, including bacteriophage and predatory bacteria, such as Bdellovibrio bacteriovorus. While bacteriophage are used as antimicrobial therapies in Eastern Europe and are being applied for compassionate use in the United States, predatory bacteria are only just beginning to reveal their potential therapeutic uses. However, predation by either predator type can falter due to different adaptations arising in the prey bacteria. When testing poultry farm wastewater for novel *Bdellovibrio* isolates on Escherichia coli prey lawns, individual composite plaques were isolated containing both an RTP (rosette-tailed-phage)-like-phage and a B. bacteriovorus strain and showing central prey lysis and halos of extra lysis. Combining the purified phage with a lab strain of B. bacteriovorus HD100 recapitulated haloed plaques and increased killing of the E. coli prey in liquid culture, showing an effective side-by-side action of these predators compared to their actions alone. Using approximate Bayesian computation to select the best fitting from a variety of different mathematical models demonstrated that the experimental data could be explained only by assuming the existence of three prey phenotypes: (i) sensitive to both predators, (ii) genetically resistant to phage only, and (iii) plastic resistant to B. bacteriovorus only. Although each predator reduces prey availability for the other, high phage numbers did not abolish B. bacteriovorus predation, so both predators are competent to coexist and are causing different selective pressures on the bacterial surface while, in tandem, controlling prey bacterial numbers efficiently. This suggests that combinatorial predator therapy could overcome problems of phage resistance.

**IMPORTANCE** With increasing levels of antibiotic resistance, the development of alternative antibacterial therapies is urgently needed. Two potential alternatives are bacteriophage and predatory bacteria. Bacteriophage therapy has been used, but prey/host specificity and the rapid acquisition of bacterial resistance to bacteriophage are practical considerations. Predatory bacteria are of interest due to their broad Gram-negative bacterial prey range and the lack of simple resistance mechanisms. Here, a bacteriophage and a strain of Bdellovibrio bacteriovorus, preyed side by side on a population of E. coli, causing a significantly greater decrease in prey numbers than either alone. Such combinatorial predator therapy may have greater potential than individual predators since prey surface changes selected for by each predator do not protect prey against the other predator.

## Abstract (precis)

With increasing levels of antibiotic resistance, the development of alternative antibacterial therapies is urgently needed. Two potential alternatives are bacteriophage and predatory bacteria. Bacteriophage therapy has been used, but prey/host specificity and the rapid acquisition of bacterial resistance to bacteriophage are practical considerations. Predatory bacteria are of interest due to their broad Gram-negative bacterial prey range and the lack of simple resistance mechanisms. Here, a bacteriophage and a strain of Bdellovibrio bacteriovorus, preyed side by side on a population of E. coli, causing a significantly greater decrease in prey numbers than either alone. Such combinatorial predator therapy may have greater potential than individual predators since prey surface changes selected for by each predator do not protect prey against the other predator.

## INTRODUCTION

Rapidly rising levels of antimicrobial resistance in Gram-negative bacterial pathogens have highlighted the urgent need for the development of alternative forms of antibacterial therapies (1), and the World Health Organization has listed several as critically urgent for new therapeutics. Many Gram-negative pathogens can be killed by a variety of bacteriophage (“phage”) and by predatory bacteria, including Bdellovibrio bacteriovorus (2, 3). Bacteriophage have been used regularly in Eastern Europe and Russia as antimicrobial therapies (4). However, the development of bacterial resistance to bacteriophage can occur rapidly both *in vitro* and *in vivo* by receptor gene mutations (5–7), leading to the requirement for, and the development of, phage cocktails for therapeutic purposes, including recent compassionate treatment use (8, 9). *Bdellovibrio* have recently been the subject of a number of *in vivo* studies to test their efficacy in animals (10–12) but have yet to be trialed for use in humans. Unlike bacteriophage, there are no known simple receptor gene mechanisms for resistance.

Bacteriophage are obligate intracellular predators that can be found in environments wherever susceptible bacteria are available; more than 95% of phage isolates described to date belong to the order *Caudovirales* or “tailed phage” (13). The tails of these phage attach to receptors on the surface of the host bacterium, including flagella (14), lipopolysaccharide (15), or outer membrane proteins (16). Due to the specific nature of the receptor for phage attachment, the host range of each phage is typically quite small, determined by the prevalence and conservation of phage receptors in bacterial populations (17). The cellular machinery of the bacterium is rapidly hijacked by the phage, after injection of the viral genome, and redirected to synthesize and assemble new phage virions that are released to start a new infection cycle (2). Host resistance against bacteriophage infection falls within four general categories: inhibition of adsorption, blocking injection of the viral genome, recognition and restriction modification of bacterial DNA, and inhibition of the transcription and replication of phage DNA (18, 19).

B. bacteriovorus predation is a biphasic process, consisting of a flagellate, rapidly swimming phase, before colliding with, attaching to, and invading Gram-negative bacteria (which can be either actively growing or in stationary phase) (20). B. bacteriovorus invade prey cells by interacting with the outer membrane, creating a pore in the outer membrane and wall, through which they enter into the prey cell periplasm, sealing the pore behind them, forming a rounded structure called a bdelloplast (20). Unlike bacteriophage, which hijack prey replication machinery for their own replication, *Bdellovibrio* invasion results in the rapid death of the prey cell (20, 21). Periplasmic *Bdellovibrio* secretes many enzymes into the prey cell cytoplasm, using the cytoplasmic contents for growth. The *Bdellovibrio* elongates, divides into multiple progeny cells, lyses the prey bdelloplast, and is released (22).

By growing intracellularly, the *Bdellovibrio* is within an enclosed niche and does not have to compete with other bacteria for resources. The only known protection against predation is the synthesis of a paracrystalline S-layer by prey cells; however, the *Bdellovibrio* is still able to prey on S-layer^+^ cells should there be any patchiness to the S layer (23). It has been observed that, in laboratory culture, not all prey bacteria are killed by *Bdellovibrio*; a small population exhibits a “plastic” resistance phenotype; when removed from predators and allowed to grow, the resulting cells are as sensitive to *Bdellovibrio* predation as the original prey population (24). Prey resistance to antibiotics does not result in resistance to *Bdellovibrio* predation, as has been shown in multiple studies looking at drug-resistant Gram-negative pathogens (25, 26). Although well known for their predatory nature, B. bacteriovorus organisms are not obligate predators; approximately one in a million *Bdellovibrio* organisms from a predatory culture can be grown axenically, prey/host independently (HI), on complex media without prey (27).

Mathematical modeling of bacterial predation is being increasingly applied to understanding predation kinetics of either bacteriophage or *Bdellovibrio*; however, modeling of predation by both types of predators on the same prey species has not yet been reported. Bacteriophage predation has been the subject of numerous studies [reviewed in references 7 and 28]), with the models becoming increasingly complex through the inclusion of the effects of the rise of prey resistance (6), altered nutrient availability, multiple bacterial species, and more (28). Modeling of *Bdellovibrio* predation is more limited, having started from the original Lotka-Volterra equations (29), via considering a delay between prey death and predator birth (30) to models that consider the bdelloplast stage as a separate population rather than just as a delay (31–34). Few papers considered decoys (33, 34), and one of these integrated experiments and adjusted the model to match the experiments (33). Other models have considered the effect of a refuge on predation (32), the effect of serum and “plastic” resistance of prey to *Bdellovibrio* on predation (31), or how predation efficiency depends on prey size and other factors (35).

Here, during sampling standing water on a poultry farm for novel *Bdellovibrio* isolates, single haloed plaques were observed on E. coli prey lawns. Within each haloed plaque were both a predatory Bdellovibrio bacteriovorus and a coisolated bacteriophage. Here, we use “prey” as a unified term that encompasses both prey for *Bdellovibrio* and host for bacteriophage, since in this work a single bacterium, E. coli, acts as both prey and host, and we are comparing the action of two different predators.

The phage genome was partially sequenced and shown to be homologous to that of a rosette-tailed-phage (RTP) (36). The RTP phage family differ in tail structure but are related to the T1 phages, the receptor for some of which is a component of the E. coli outer membrane, and host resistance is reported to arise frequently (36).

Our experimental analysis of predation kinetics revealed that when both predators were combined in one culture with E. coli prey, complete prey lysis was achieved in 48 h. This was in contrast to cultures containing either of the single predators where prey remained; with phage alone the remaining prey were phage resistant, whereas with *Bdellovibrio* alone a subpopulation of prey remained but no acquisition of genetic resistance occurred. Mathematical modeling of this experimental system revealed that both the phage resistance and the plastic resistance to *Bdellovibrio* predation arose in the E. coli prey population and that the two predators were most likely acting independently and competitively rather than cooperatively. We show here that two bacterial predators can be coisolated from the environment, coexist in lab cultures, and when applied in combination can result in greater killing of the prey bacterial population than by either predator alone, suggesting that *Bdellovibrio*-phage combinations may be a successful approach toward therapeutic antibacterials.

## RESULTS

### Isolation of environmental *B. bacteriovorus* and associated bacteriophage.

**FIG 1:**
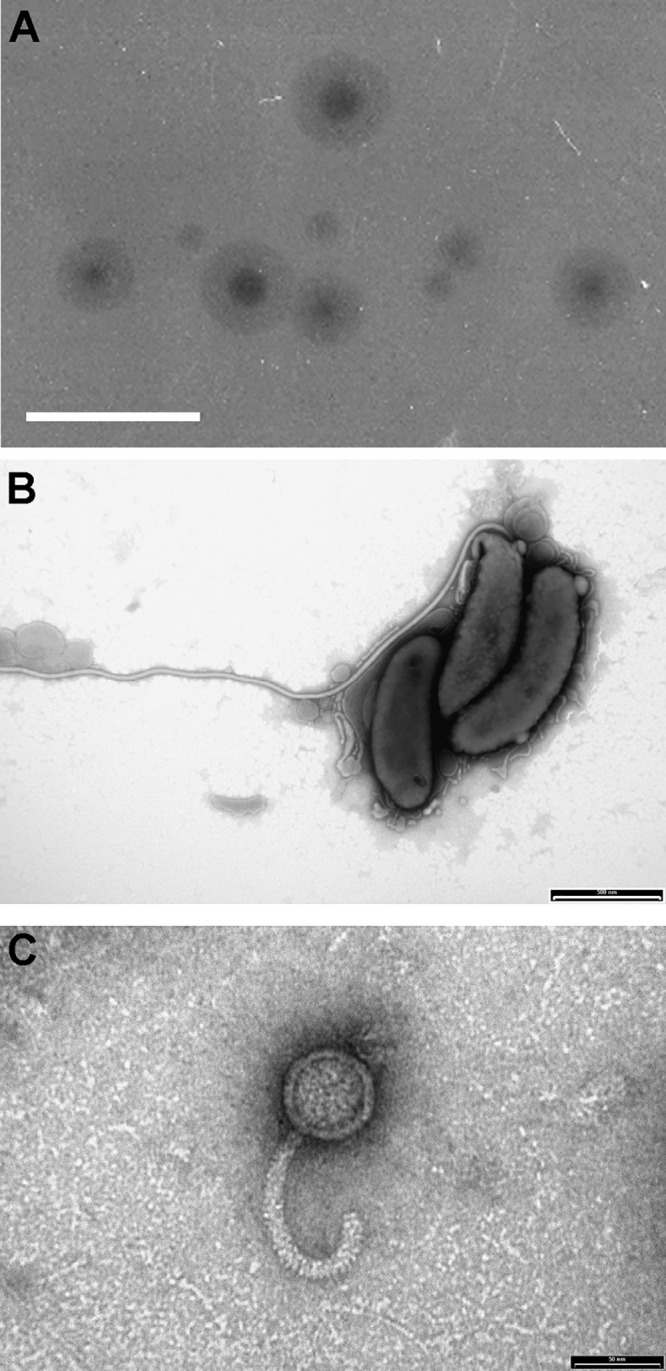
Unique haloed plaque morphology from which the coisolated novel B. bacteriovorus angelus and bacteriophage halo were identified by electron microscopy. (A) Haloed plaques containing both B. bacteriovorus angelus and bacteriophage halo on lawns of E. coli in YPSC double-layer agar plates. Scale bar, 1 cm. (B) Electron microscopy of B. bacteriovorus angelus, stained with 0.5% URA (pH 4.0). Scale bar, 500 nm. (C) Electron microscopy of a 0.22-μm filtrate of a predatory culture, showing the presence of phage particles with curved tails resembling bacteriophage RTP. Phage were stained with 0.5% URA pH 4.0. Scale bar, 50 nm.

When isolating *Bdellovibrio* from 0.45-μm-pore filtrates of standing water on a poultry farm, one isolate rapidly lysed offered E. coli lab cultures and repeatedly produced plaques with large “halos” around them on prey lawns (Fig. 1A). These plaques contained characteristic small, highly motile B. bacteriovorus-like bacteria (Fig. 1B) and “bdelloplasts,” i.e., infected E. coli prey cells containing live B. bacteriovorus. Sequencing and alignment of the 16S rRNA gene amplified from predatory *Bdellovibrio* purified from a single isolated “haloed” plaque showed that the *Bdellovibrio* was a member of the B. bacteriovorus species, and its 16S rRNA sequence (GenBank accession no. GQ427200.1) was 99% identical to that of the type strain HD100 (37). Therefore, the isolated *Bdellovibrio* was named B. bacteriovorus angelus due to the initial haloed appearance of the plaques from which it was isolated.

Predatory cultures derived from individual “haloed” plaques, when filtered through 0.22-μm filters, which retain B. bacteriovorus, were found to contain an agent that lysed E. coli, giving different cell debris (without the rounded bdelloplasts). The concentrated filtrate showed several prominent protein bands on SDS-PAGE (see Fig. S1A in the supplemental material). One of these bands (∼30 kDa) was found, by using matrix-assisted laser desorption ionization/quadrupole time-of-flight mass spectroscopy (MALDI-QToF MS) (Fig. S1B), to contain five peptides which were homologous to the 34 kDa protein RTP27 (GenBank accession no. CAJ42231.1) of a rosette-tailed phage (RTP) of E. coli (36). Simultaneous electron microscopy of the 0.22-μm filtrate revealed many phage particles with curved tails that resembled RTP, without such a pronounced rosette on the tail (Fig. 1C). The phage was given the abbreviated name “halo” and the 46-kDa double-stranded DNA phage genome was purified, and 7 kb of it was sequenced (GenBank accession no. GQ495225.1; the bacteriophage halo was named RES2009a) and compared in BLAST to other phage genomes. The best matches were to phage genomes belonging to the “rtpvirus” genus, including the characterized RTP phage (EMBL accession no. AM156909.1) (36). The phage halo was plaque purified away from the B. bacteriovorus, using kanamycin-resistant E. coli as prey (as B. bacteriovorus angelus was found to be kanamycin sensitive, as is the type strain HD100), and so was inhibited from predatorily replicating in the kanamycin-resistant E. coli in the presence of the antibiotic).

Thus, B. bacteriovorus angelus and bacteriophage halo had been coisolated, from the same environment, via single “haloed” plaques in bacterial prey lawns in which both predators were preying, side by side, upon the same offered E. coli population, and thus it is possible that they prey similarly in the natural environment.

### *E. coli* resistance to bacteriophage halo occurred rapidly.

**FIG 2:**
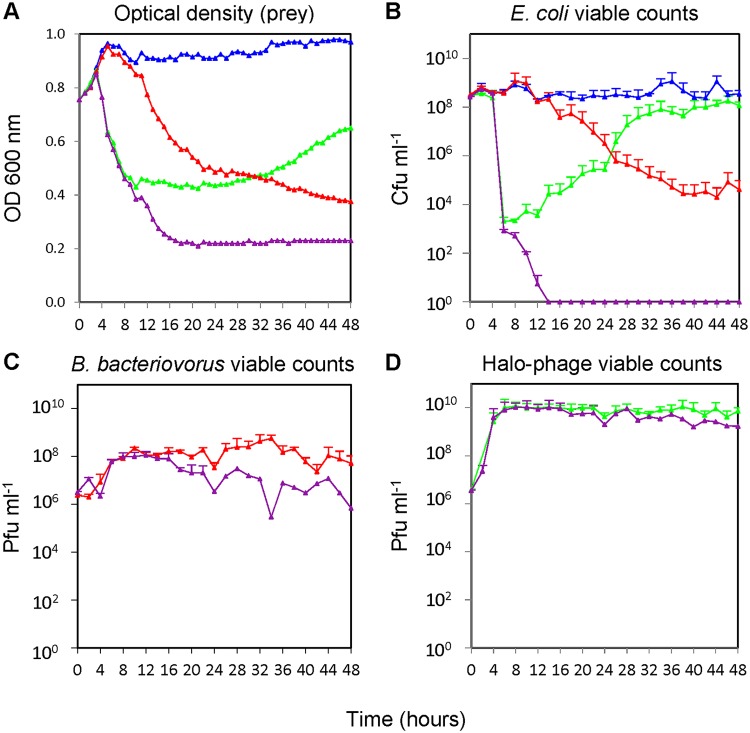
Kinetics of predation. Measured over 48 h on late-log-phase E. coli S17-1 by bacteriophage halo alone (green), B. bacteriovorus HD100 alone (red), and both bacteriophage halo and B. bacteriovorus HD100 combined (purple) versus E. coli plus buffer control (blue). (A) E. coli measured as the OD_600_ (B. bacteriovorus organisms are too small to register at OD_600_). (B) E. coli viable counts. (C) B. bacteriovorus HD100 enumeration by plaque counts. (D) Bacteriophage halo enumeration by plaque counts.

Rapid phage resistance was observed in E. coli S17-1 cultures that were preyed upon by the bacteriophage halo alone, with a persistent level of E. coli remaining after 16 h of infection (see Fig. 2B for an example from later growth experiments). Two independently derived phage-resistant E. coli cultures (F and G) were isolated by plating out the remaining E. coli prey cells from these 16-h cultures, which were preyed upon by the phage alone. The two isolates were verified as being phage resistant by being tested for phage predation again. Genome sequencing of each isolate, alongside the original E. coli S17-1 strain used in the experiments, was performed to identify the mutations that resulted in phage resistance. This revealed (Table 1) that two different IS4 transposase insertions had occurred and been selected for in the genomes of resistant strains F and G within the same gene, encoding the ligand-gated outer membrane porin FhuA responsible for ferric hydroxamate uptake through the outer membrane (36). The FhuA protein is known to act as a receptor for other phages and is likely to be the receptor for phage halo (38).

**TABLE 1:** Mutational changes present in the genome sequences of the bacteriophage resistant mutants

Accession no.	Gene product	Nucleotide position	Change in coding region	Reading frame change	Mutant isolate(s)^*a*^
FGH86_13085	KdbD TCS sensor histidine kinase	2690204	G-to-A substitution	D571N	F and G
FGH86_16680	FhuA ferric hydroxamate transporter/phage receptor	3364483	IS*4*-like insertion	Inactivation	F
FGH86_16680	FhuA ferric hydroxamate transporter/phage receptor	3365489	IS*4*-like insertion	Inactivation	G
FGH86_19640	Paraslipin	4005457	C-to-T substitution	S25F	F and G
FGH86_19645	Ribosome release factor	4005510	A-to-G substitution		F and G

a Mutations in mutants F and G are presented relative to the reference chromosome sequence of E. coli S17-1 (CP040667).

The two halo-phage-resistant E. coli derivatives grew at rates similar to those of the parental E. coli S17-1 strain. Using the phage-resistant E. coli as prey in lawns in overlay plates allowed for plaque formation by, and subsequent purification of, the B. bacteriovorus angelus isolate away from the phage (Fig. S2A), since phage resistance did not confer any resistance to predation by B. bacteriovorus.

We also verified (data not shown) that B. bacteriovorus is not susceptible to lytic or lysogenic infection by bacteriophage halo in two tests. First, we used host-independent derivatives of both B. bacteriovorus angelus and HD100 (isolate HID13 [21]) as prey in lawns onto which bacteriophage halo was added. No zones of clearing were observed, even after prolonged incubation. Second, after the addition of bacteriophage to liquid cultures of pure attack-phase B. bacteriovorus angelus, or HD100, no evidence of phage infection was seen when the samples were observed microscopically or enumerated. Thus, B. bacteriovorus itself is not susceptible to the bacteriophage halo during either predatory or prey-independent lifecycles.

### Experimental predation by combined *B. bacteriovorus* HD100 and halo phage predators eradicates *E. coli* prey unlike single predators.

To test the effects of predation by the two predators on a single prey population at the same time, the kinetics of predation by equal numbers of phage alone, B. bacteriovorus alone, and B. bacteriovorus plus phage on E. coli S17-1 were measured alongside an E. coli with buffer control (Fig. 2) using the methods detailed below. We had found no specific association between the phage and the environmental B. bacteriovorus coisolate since mixing the purified halo phage and pure B. bacteriovorus angelus or B. bacteriovorus HD100 suspensions together both reconstituted haloed plaques on a lawn of E. coli prey. Having noted that the predation rates in liquid cultures of each of the two B. bacteriovorus strains, angelus and HD100, were the same but that HD100 forms larger (and hence more visible and countable) plaques, we used the HD100 strain in predation kinetics studies on E. coli with or without the phage.

Because phage are usually grown in log-phase prey cultures in broth and B. bacteriovorus is usually grown on stationary-phase prey in calcium HEPES buffer, a “compromise” late-log-phase E. coli prey, at a starting optical density at 600 nm (OD_600_) of 0.75, was used with a mean initial E. coli population of 2.9 × 10^8^ CFU/ml. Deliberate inclusion of an equal volume of background YT medium used for the E. coli preculture in calcium HEPES buffer gave a low-nutrient environment, which allowed for E. coli viability throughout the 48-h test period (Fig. 2B).

The overall kinetics of the 48-h experiments were monitored by measuring the OD_600_ (Fig. 2A) and viable counts (Fig. 2B to D), which indicated that, during the first 24-h period, E. coli was killed more slowly by B. bacteriovorus than when preyed upon by both B. bacteriovorus and bacteriophage halo together (Fig. 2B). When incubated solely with the bacteriophage halo, the E. coli numbers decreased rapidly, reaching the lowest prey density of 2.1 × 10^3^ CFU/ml at 6 to 8 h; the E. coli population then began to increase due to the increase in phage-resistant cells within the prey population (Fig. 2B). Interestingly, when the prey were incubated with both the phage and the B. bacteriovorus, this increase in prey numbers did not occur. Instead, the E. coli population was eradicated after 14 h, dropping to below detectable numbers (<10 CFU/ml) (Fig. 2B). The phage and B. bacteriovorus population numbers were lower (by 10- and 100-fold, respectively, at the 48-h time points) in the combined culture, likely due to the reduced numbers of prey available to each predator population (Fig. 2C and D). It is noteworthy that adding an equal number of 5 × 10^6^ PFU/ml of the other predator, each with the potential to kill and remove an E. coli cell from the available prey pool, caused 10-fold less reduction in phage numbers than in B. bacteriovorus numbers. This may be due to the more rapid kinetics of E. coli predation by phage versus the slower kinetics of killing by B. bacteriovorus. Since the emergence of genetic or plastic resistance, respectively, to the two different predators would be expected to have a major effect, we modeled these processes mathematically to investigate them further.

### Mathematical modeling of copredation.

**FIG 3:**
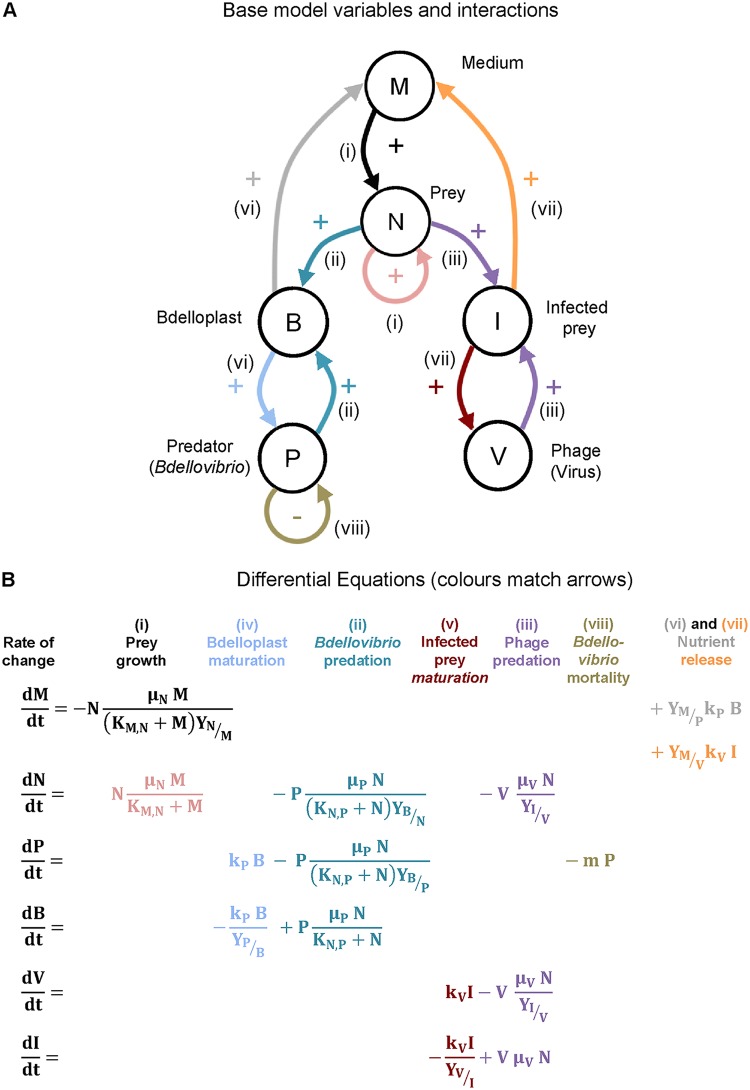
Base model with one prey type. (A) Diagram of the model variables (populations and chemicals) in circles and their positive or negative interactions. The arrow colors match the colors of the terms in the equations in panel B, and the roman numerals refer to the list of processes in the text. (B) Set of differential equations defining the base model.

Modeling started from a one-prey and one-predator model (35). A bacteriophage was added as a second predator to build the base model of the experimental system (Fig. 3). This base model has one (E. coli) prey type (N) and two consumers of the prey, the predator B. bacteriovorus (P) and the virus bacteriophage halo (V). Both attack and enter the prey to form a distinct stage, thereby removing prey and predator from their respective populations. When B. bacteriovorus enters the prey, a bdelloplast (B) is formed. When the phage infects the prey, an infected prey (I) is formed. Upon lysis of B or I, resources enabling regrowth of prey called M (for medium) are released, together with the respective predator offspring.

The combined resource M is needed because the experimental data show regrowth of E. coli during halophage predation (Fig. 2B). Altogether, the base model (Fig. 3A) has 6 variables shown as circles. Processes are shown as arrows and terms of the equations in Fig. 3. These are (i) prey growth by consumption of medium, (ii) predation of prey by available B. bacteriovorus to yield the bdelloplast, (iii) predation by free bacteriophage halo (virus) to yield the Infected prey, (iv) maturation (replication and development) of B. bacteriovorus within the bdelloplast, (v) maturation of the bacteriophage (virus) within the Infected prey, (vi) lysis of bdelloplast which yields free replicated B. bacteriovorus and releases nutrients which replenish medium, and (vii) lysis of infected prey, which yields free virus and also releases nutrients that replenish medium. The nutrients remaining were not sufficient to produce further whole-progeny B. bacteriovorus or more phage but will be a small residue of what did constitute the original prey cell since most of the nutrients were used in producing B. bacteriovorus or phage progeny. As mentioned above, the medium does allow some limited growth of the prey.

We also included (viii) mortality for B. bacteriovorus, as this was evident from Fig. 2C and is well known from the literature (33, 39). We did not include mortality for E. coli and the halophage since the data showed no evidence for this during the 48-h experimental time period (there was no statistically significant trend [Fig. 2B and D]).

**FIG 4:**
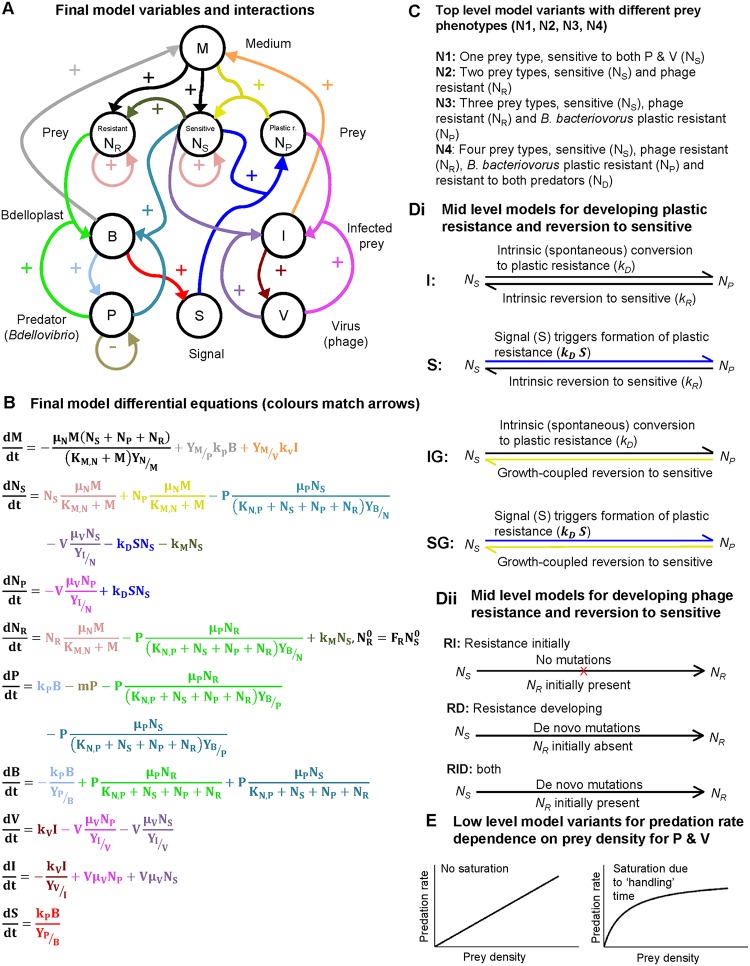
Final model and model variants. (A) Diagram of the final model variables (populations and chemicals) and their positive or negative interactions. The arrow colors match the colors of the terms in the equations in panel B. (B) Set of differential equations defining the final model. (C) Top-level model variants with different prey phenotypes (models N1, N2, N3, and N4). (D) Midlevel model variants. (Di) Methods of development of plastic resistance to B. bacteriovorus; (Dii) methods of development of phage resistance. (E) Low-level model variants. The predation rate either saturates at high prey densities or does not (it can differ between B. bacteriovorus and phage).

**FIG 5:**
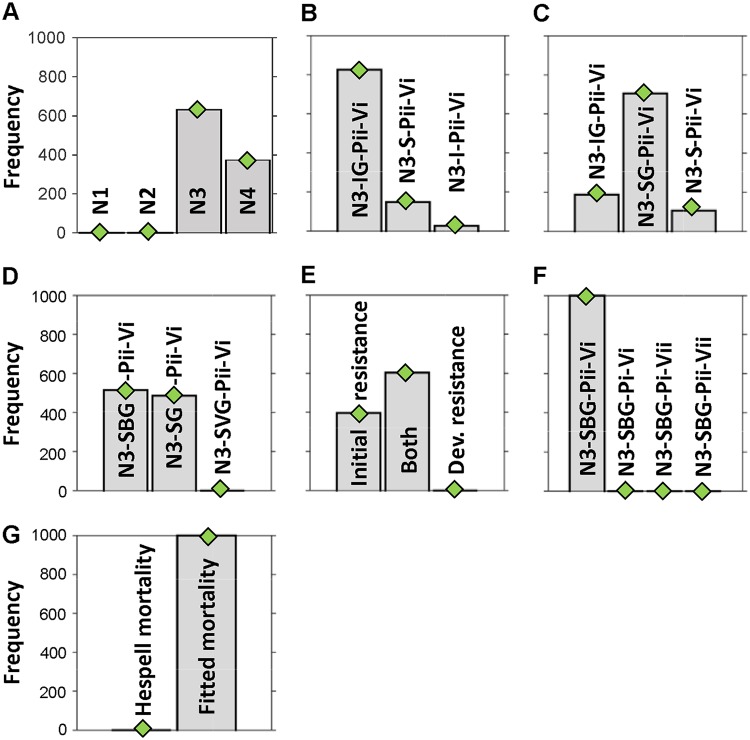
Hierarchical model selection process. This infers which model variants from Fig. 4 are best supported by the data (frequency of a variant winning out of 1,000). (A) Competition of models with different number of prey phenotypes. N1, one prey type sensitive to both predators (N_S_); N2, two prey types, N_S_ and phage-resistant prey (N_R_); N3, three prey types, N_S_ and N_R_ and prey with plastic phenotypic resistance to B. bacteriovorus (N_P_); N4, four prey types, N_S_, N_R_, N_P_, and prey with dual resistance (N_D_). (B) Competition of models with different ways of converting between sensitive prey (N_S_) and plastic-resistant prey (N_P_) but the same saturating B. bacteriovorus attack rate (Pii) and nonsaturating phage attack rate (Vi). N3-IG-Pii-Vi, N_S_ intrinsically (spontaneously) converts to N_P_ and back conversion is coupled to growth. N3-S-Pii-Vi, N_S_ conversion to N_P_ is triggered by a signal and back conversion is spontaneous. N3-I-Pii-Vi, spontaneous conversion both ways. (C) The combined variant from panel B is in the middle and its “parent” variants are on either side. N3-SG-Pii-Vi, N_S_ conversion to N_P_ is triggered by a signal, and back conversion is coupled to growth. (D) Model variants, derived from the combined model in panel C but differing in the way the signal is produced. N3-SBG-Pii-Vi, signal derives from interaction of prey and B. bacteriovorus only. N3-SG-Pii-Vi, signal derives from interaction of prey with both predators. N3-SVG-Pii-Vi, signal derives from prey interaction with virus (phage) only. (E) Different ways of generating phage resistance. Phage-resistant prey were already present initially or prey developed resistance *de novo* or both. (F) Model variants, based on N3-SBG from panel D but differing in attack rate saturation. Pii, B. bacteriovorus attack rate saturates at high prey density (whereas Pi does not saturate), likewise with Vii and Vi for the virus (phage). (G) Mortality of B. bacteriovorus (phage assumed to be stable) was either set to that of Hespell et al. (39) or fitted by the ABC-SMC method. Less decisive competitions (B to D) were repeated 10 times (see Fig. S6).

From this base model (Fig. 3), we generated a family of related models, adding additional variables and processes step by step and testing different mechanisms for the transitions between entities (Fig. 4). We then used Bayesian inference to select, in several stages, the model variant that best fitted the population dynamics observed in the experiments (Fig. 5; see also Fig. S6 demonstrating reproducibility). A full description of the model variants and the approximate Bayesian computation process for model selection and parameter inference is given in supplemental material.

Competing the top-level model variants with one, two, three, or four prey types (Fig. 4C) gave clear results (Fig. 5A). The model variant N1 with prey sensitive to both predators (N_S_) and variant N2 with only N_S_ and bacteriophage-resistant prey (N_R_) were not supported by the experimental data at all. The variant N3 with N_S_, N_R_, and prey exhibiting the “plastic” phenotypic resistance to B. bacteriovorus predation (N_P_) was best supported by the experimental data, while variant N4, including the doubly resistant prey (N_D_), was less supported (Fig. 5A). N3 and N4 are nested models with the same number of parameters, so fitting variant N4 is not intrinsically more difficult. Using the parameter values generated by fitting either of variant N3 or variant N4 predicted similarly low levels of doubly resistant prey at the end of the experiment when applied to the equations of variant N4. Variant N4 fitted to all data predicted 0.26 CFU/ml, while the same variant using parameters from fitting variant N3 to all data predicted 0.0084 CFU/ml. Both are well below the detection threshold in the experiments (10 CFU/ml). Variant N4 predicts double resistance to occur, albeit at a very low level; however, the data could not provide information to constrain this density. Due to these considerations and the aim to choose the minimal adequate model, the N3 model variant was selected for further study.

After selecting this three-prey type N3 model, we tested various submodels based on different ways in which the sensitive prey type converts to the type with plastic phenotypic resistance to B. bacteriovorus and back (Fig. 4Di). The simplest assumption is that forward and backward conversion occur spontaneously at certain rates, without any external triggers (intrinsic conversion both ways, variant I). This was not supported by the data (Fig. 5B). Another model variant replaces the intrinsic back conversion with a growth-coupled conversion (variant IG). This variant was well supported by the data. A third variant replaces the intrinsic conversion by a signal-triggered conversion to plastic resistance (variant S). At this initial stage in the modeling, the signal was assumed to be generated by the lysis of bdelloplasts and phage-infected cells. Plastic resistance has been previously described (24) as developing to B. bacteriovorus in predatory cultures due to (as yet unidentified) molecular signals changing prey metabolism/development, but it is not due to genetic changes in the prey since when those prey are grown in new cultures and rechallenged with B. bacteriovorus they are susceptible once more (21). This variant S had some support from the data (Fig. 5B). Hence, we tested whether a combination of the two supported variants would fit better. This combined variant SG, with signal triggered conversion to plastic resistance plus growth-coupled back conversion, was better supported by the data than its parental variants (Fig. 5C).

Following this, we compared variants where the source of the signal was interaction of prey with phage only, with B. bacteriovorus only, or with both (Fig. 5D). Since there was no evidence for phage involvement and the two variants with B. bacteriovorus involvement were about equally supported, we concluded that B. bacteriovorus interaction with prey was sufficient to generate the signal for plastic resistance.

Likewise, we looked in the model at different ways in which the phage-resistant prey arises (Fig. 4Dii). We compared the simpler submodels where some phage-resistant prey is already present at the beginning of the experiment, as in the classic fluctuation test of Luria and Delbrück (40), or only develop as *de novo* mutations during the experiment with the combined submodel that had both preexisting and *de novo* mutations. This combined model variant was best supported by the data, and *de novo* developing mutations alone are insufficient to explain the data (Fig. 5E).

### Modeling predation rate saturation.

After finding the “best” or most appropriate model variant for prey-type conversions, we looked at the low-level model variants (Fig. 4E), where details of the model are varied but not the number of prey types and their conversion. One such detail is whether the predation rate saturates at a higher prey density or not (Fig. 4E). Only the variant assuming no saturation of the predation rate for the phage but saturation of the predation rate for B. bacteriovorus was supported by the data (Fig. 5F). This does not mean that phage predation would not saturate at higher prey densities than we investigated in this study but that the bacterial predator saturates at lower prey densities than the phage (see the parameters in Table S1 in the supplemental material). This is expected since the longer the prey “handling time” for a predator, the more its response will saturate when prey becomes abundant (41). It is well known that B. bacteriovorus takes longer to attach and enter its prey periplasm than phage (20), and our results support this (42). Lack of saturation facilitates the observed rapid initial prey killing by phage (Fig. 2). We did not consider saturation effects at high phage densities in this study because there was little information in the data from experiments that concentrated on later time points and the rise of phage resistance to parameterize phage saturation (there is only a brief interval with high phage density while sensitive prey are available, see Fig. 2B and D). We did, however, model different initial prey densities, as shown in Fig. S8 (see below).

### The final model shows effective side-by-side action of dual predators.

**FIG 6:**
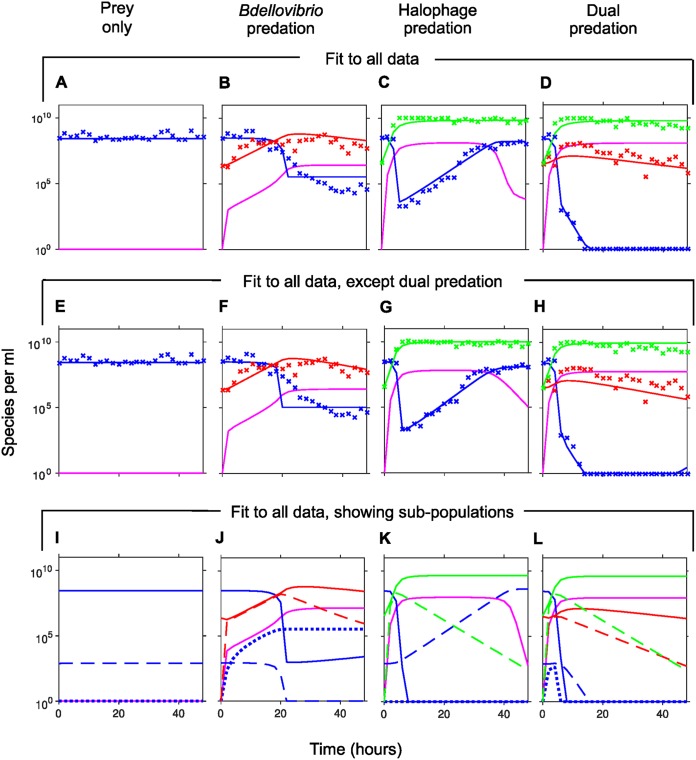
Comparison of experimental data (mean values) with fits of the best model variant (from Fig. 5). The model was fitted using either all experimental data (A to D) or all data without dual predation (E to H) and then used to predict the outcome of dual predation (shown in panel H). The parameter values for each case are given in Table S1 in the supplemental material. Experimental data are indicated by symbols; lines represent model simulations. Colors in panels A to H: blue, E. coli prey; red, B. bacteriovorus; green, bacteriophage halo; pink, medium (not experimentally measured). (I to L) Dynamics of the subpopulations of prey and predators predicted by the model that was fitted to all data, corresponding to panels A to D. Colors and lines in panels I to L (as they relate to models in Fig. 3 and 4): blue, E. coli prey (solid line, susceptible prey [N_S_]; dotted line, plastic-resistant prey [N_P_]; dashed line, bacteriophage-resistant prey [N_R_]); red, B. bacteriovorus (solid line, free B. bacteriovorus [P]; dashed line, bdelloplasts [B]); green, bacteriophage halo (solid line, free bacteriophage [V]; dashed line, bacteriophage-infected cells [I]); pink, medium.

The final, most appropriate model variant was then fitted to all the data (Fig. 6A to D). We explain in the supplemental material how we used principal component analysis (PCA) to objectively select a typical parameter set out of the hundreds of accepted fits. The final model fits the prey dynamics well, apart from the exact kinetics of the decline of prey in the presence of B. bacteriovorus as the only predator (Fig. 6B) where prey density does not drop as gradually in the model as in the experiments. Despite trying many variants of prey type conversions, we could not find any variant that would give a better fit to this more gradual decline of prey without making the fit to other parts of the data much worse, so Fig. 6A to D shows the best fit we could obtain.

We also compared the fit of this final model to all data (Fig. 6A to D) with the fit of the same model to all data, excluding that from two predators acting on one prey (Fig. 6E to H). The two fits are almost the same. This means that the experimental results can be explained without invoking any direct interactions between the two predators.

### Dependence on initial densities.

To understand the dependence of predation success on the initial densities of prey and predators, we used the model to predict the outcome if we varied one population at a time, increasing as well as decreasing initial densities 10-fold (Fig. S8). The time series of the three related traces (10-fold lower, normal, and 10-fold higher initial densities) showed similar qualitative behavior for cases with prey only and prey with a single predator. Here, the three traces either converged in the end, or their separation was less than the 10-fold initial separation. Prey could survive during dual predation if (i) the initial density of prey was too high, (ii) the initial density of B. bacteriovorus was too low, or (iii) the initial density of the phage was too high (Fig. S8F to H). The model can thus identify suitable densities of the predators to add for effective predation.

### Modeling reveals interactions of subpopulations of predators and prey.

The modeling allowed insights into the different subpopulations that comprised the observed total bacterial populations (Fig. 6I to L). In the simulated B. bacteriovorus-only predation, the B. bacteriovorus population is evenly split between free B. bacteriovorus and bdelloplasts from 2 to 20 h. Afterward, the bdelloplasts decline exponentially, while free B. bacteriovorus numbers increase a little (due to progeny release from bdelloplasts) and then decline again due to their mortality (Fig. 6J). Both the fully susceptible and phage-resistant prey populations plummet at 20 h, when the plastic-resistant prey has reached a plateau (Fig. 6J). In the simulated phage-only predation, sensitive prey rapidly dropped in the first 6 h; afterward, the phage-resistant prey increased exponentially until reaching a plateau (Fig. 6K). In the simulated dual predation, the phage is mostly responsible for the rapid drop of the susceptible prey and the removal of the intermittently arising plastic B. bacteriovorus resistant prey, while B. bacteriovorus is responsible for the removal of the phage-resistant prey. All three prey populations are eradicated by the two predators together (Fig. 6K).

## DISCUSSION

When attempting to isolate *Bdellovibrio* strains from environmental sources, a sample of chicken farm wastewater gave haloed plaques on lawns of E. coli due to the combined predation by the new strain of B. bacteriovorus, which we named angelus, and an RTP-like bacteriophage, which we named halo. The combined predation was also produced by the addition of bacteriophage halo to lab strain B. bacteriovorus HD100. We combined both experimental and mathematical modeling approaches to unravel the dynamics of this combinatorial predation, showing that a combination of two microbial predators eradicated a single pathogenic bacterial species under conditions when each alone did not. The modeling suggested that B. bacteriovorus killed all the phage-resistant prey types and the phage halo killed all the plastically B. bacteriovorus-resistant prey. This suggests that combinatorial predator therapy may be one approach to tackle the problem of phage resistance in phage therapy treatments.

Although found coassociated in nature, the RTP-family phage halo did not attach to, lyse, or lysogenize the B. bacteriovorus but was found to prey alongside it on E. coli in experimental lawns, producing the haloed plaques.

There were several possibilities for how the combined predators were behaving in the mixed cultures: (i) were they acting independently on the prey, in competition with each other at overlapping receptor sites; (ii) were the phage aiding in some way predation by the *Bdellovibrio* or—vice versa—were the phage acting as an opportunistic passenger; (iii) or were there subsets of the prey population that were susceptible to predation by each? The mathematical modeling allowed investigation of this beyond experimental limits. The model selection results revealed the presence of three subsets of the prey population, those susceptible to both predators and those resistant to predation by either the phage or the bdellovibrio.

The final model gave a good fit to the copredation experimental data. Moreover, when fitted to just the data sets containing the prey only and the single predators, the resulting parameter values gave a very similar fit to the experimental data for the combined predation conditions. Since the final model does not contain any terms for direct interactions between the two predators, combined with the fact that fitting to single predator data predicts the combined predation results, we conclude that the two predators act independently.

One question that did remain was why did we isolate haloed plaques from the environment which contained both predators if they can operate independently? Clearly, during our dual predation experiments, a final yield of c1 × 10^10^ phage were present from a prey population which yielded c1 × 10^6^ B. bacteriovorus PFU, so the phage were in 10,000-fold excess. The high phage abundance was probably the reason for their presence in each plaque. The rapid accumulation of phage-resistant populations of E. coli, preyed upon by phage, provides no barrier to B. bacteriovorus predation and so does not prevent cooccurrence.

Purification of each predator made it possible to study their individual and combined effects in ways not possible in other studies (43). Employing a low-nutrient environment allowed predation by each predator and allowed sustained viability of the E. coli population over the 48 h of investigation. Experimental predation by the *Bdellovibrio* alone resulted in a gradual decrease in prey numbers from 1.2 × 10^9^ CFU/ml to a minimum of 2.0 × 10^4^ CFU/ml (Fig. 2). This is consistent with other reports of *Bdellovibrio* predation on a variety of different prey species, where complete killing of the prey population was not observed (26, 31, 33). The modeling revealed that a subpopulation of prey arose that would exhibit a “plastic” resistance to *Bdellovibrio* predation, a form of resistance that is not genetically encoded and is also not passed to daughter cells, consistent with the “plastic” resistance phenotype previously reported (24). It had previously been hypothesized (24) that this resistance would arise due to the release of a molecular signal from the lysis of the bdelloplast, and the modeling supports such a mechanism. This “plastic” resistance may pose a problem if considering the therapeutic application of *Bdellovibrio* (3), since it may limit the reduction of pathogen numbers, although the immune system has been shown to act synergistically *in vivo* (12). In addition, physiological state of prey (leading to plastic resistance or not) may be different in the *in vivo* growth conditions. Our modeling predicts that, in a dual predation setting, the balance between applied predator numbers is important and that adding sufficient but not excess phage with B. bacteriovorus gives the best outcome.

Predation by the phage alone resulted in a 10-fold larger (but transient) decrease of the prey population to 2.1 × 10^3^ CFU/ml (seen at 6 h, Fig. 2B), before phage-resistant prey growth resulted in a final prey population at 48 h similar to the starting population. The model assumed the presence of a small fraction of phage-resistant prey at the beginning of the experiment; the median value of this fraction was 2.6 × 10^−6^ after fitting (see Table S1 in the supplemental material). This is similar in order of magnitude to previously reported values for E. coli (5, 40). The model evaluations indicated that the rise in bacteriophage-resistant prey resulted both from the growth of this initial, resistant population and spontaneous mutations arising in members of the initially phage-sensitive prey population. Both were selected for during the time course of the experiment. Replication of the phage-resistant prey resulted in the production of phage-resistant progeny, consistent with resistance being the result of genetic mutation. Sequencing of the phage-resistant genomes points to the absence of the ferric hydroxamate uptake, FhuA, protein as the reason for E. coli resistance to phage halo. This mutation would have little fitness effect in the iron-containing environment of our experimentation and given additional routes of iron uptake by E. coli.

The most noteworthy result of our study was the eradication of E. coli prey (reduction below detectable levels of <10 cells/ml) when preyed upon by both the B. bacteriovorus and the phage together (Fig. 2). The modeling revealed that the two predators were not interacting directly with each other since the experimental results could be recapitulated by the model using the data from the individual predators, without the need for the inclusion of any terms for direct interactions between predators. This suggests the potential for this phenomenon to be replicated for other combinations of Gram-negative prey, B. bacteriovorus, and prey-specific bacteriophage, something that should be further investigated (beyond the scope of this paper). Such combinatorial predator therapy could be considered as a future alternative antibacterial treatment reducing bacterial numbers to lower levels than achievable with single predators alone and reducing the selection for single predator-specific resistance.

## MATERIALS AND METHODS

### Bacterial strains, maintenance, and isolation.

E. coli S17-1 (44) prey were grown for 16 h in YT broth (45) at 37°C with shaking at 200 rpm to late log phase for use in predatory *Bdellovibrio* cultures (see below for predation kinetics description). B. bacteriovorus predatory cultures were set up as previously described and consisted of a mixture of calcium HEPES buffer, E. coli culture, and a previous B. bacteriovorus culture in a 50:3:1 (vol/vol/vol) ratio (45) at 29°C with shaking at 200 rpm. Where stated, the B. bacteriovorus type strain HD100 (37, 46) was used for comparison. Host-independent (HI) B. bacteriovorus was grown as described previously (45, 47), the HD100 derivative HID13 was described previously (21), and the angelus HI strain was obtained as part of this study.

B. bacteriovorus strain angelus and bacteriophage halo were coisolated using E. coli S17-1 as prey on YPSC double-layer agar plates as described previously (45). The bacteriophage halo was purified from the mixed phage-B. bacteriovorus cultures by growing the phage on E. coli S17-1 containing the plasmid pZMR100 (48) to confer resistance to kanamycin, which was added at 50 μg/ml, killing the kanamycin-sensitive B. bacteriovorus angelus, using repeated rounds of plaque purification on YPSC overlay plates (45, 49). Phage-resistant E. coli S17-1 was obtained by plating E. coli cells remaining in pure bacteriophage halo infection cultures and screening the resultant isolates by the addition of bacteriophage halo. These phage-resistant E. coli (strains F and G) were used to purify the B. bacteriovorus angelus from the originally mixed phage and B. bacteriovorus cocultures, again using rounds of plaque purification. The resulting purified B. bacteriovorus angelus produced small plaques (smaller than those produced by the type strain HD100 under matched conditions) on both the phage-resistant and the original phage-sensitive E. coli.

### *Bdellovibrio* DNA purification and 16S rRNA sequencing.

To phylogenetically characterize the pure *Bdellovibrio* strain isolated in the coculture, *Bdellovibrio* genomic DNA was purified from 0.45-μm filtered, host-dependently grown (before and after separation from the associated phage) and unfiltered host-independently grown B. bacteriovorus angelus using a Genelute bacterial genomic DNA kit (Sigma) according to the manufacturer’s instructions. The full-length 16S rRNA gene was amplified from a total of 11 individual genomic DNA samples using Phusion high-fidelity polymerase (Finnzymes) according to the manufacturer’s guidelines using the general bacterial primers 8F (50) and 1492r (51). Purified PCR products were sent for sequencing at MWG Biotech, Ltd., and the full-length double-stranded sequence was aligned to that of the B. bacteriovorus type strain HD100 (37).

### Phage preparation and protein identification.

Phage preparations were made by addition of bacteriophage halo (purified as described above and in Results) to a mid-log-phase culture of E. coli S17-1 (pZMR100), followed by incubation at 29°C. When the OD_600_ of the culture dropped to half that of the starting OD, chloroform was added, and the phage particles were collected using PEG precipitation, as described for lambda phage (52).

Phage preparations were run on standard 12.5% acrylamide SDS-polyacrylamide gels (53) to examine their protein content; a single band was excised and analyzed by MALDI-QToF MS, and the resulting peptide reads compared to existing sequences in the NCBI databases for the most significant hits.

### Phage and prey genomic DNA purification and sequencing.

Bacteriophage halo genomic DNA was extracted from the phage preparations described above using a Qiagen Lambda Maxi kit (Qiagen) according to the manufacturer’s instructions from step 6 to step 15. Harvested DNA was resuspended in a final volume of 1 ml of 10 mM Tris and 1 mM EDTA (pH 7.5). Restriction-digested fragments of phage genomic DNA were cloned into pUC19 (54) and sent for sequencing at MWG Biotech, Ltd., using the standard pUC19 primers M13uni(-21) and M13rev(-29). To complete the phage sequence contig, unsequenced regions of cloned fragments were PCR amplified using KOD high-fidelity DNA polymerase (Merck Millipore) and purified PCR products were sent for sequencing. A 7-kb contig of phage genomic DNA was fully sequenced, compared to other phage genomes by DNA and protein BLASTs at NCBI, and deposited in GenBank under accession number GQ495225.

E. coli S17-1 genomic DNA was prepared using a Sigma GenElute bacterial genomic DNA kit (Sigma-Aldrich Co., St. Louis, MO), from 16-h overnight cultures of wild-type and phage-resistant strains F and G. The MinION and Illumina HiSeq platforms were used to sequence the genome of E. coli S17-1 (4,772,290 nucleotides). Long-read sequences from the MinION were used as a scaffold for Illumina data consisting of 4.6 million paired-end sequence reads with lengths of 250 bp. Sequence assembly was performed using CLC Genomics Workbench, version 11.0.1 (Qiagen, Aarhus, Denmark). The genome sequence is available under GenBank accession number CP040667. Phage-resistant genome sequences were assembled using the E. coli S17-1 chromosome as the template from Illumina HiSeq data composed of 0.8 and 3.5 million paired-end sequence reads of 250 bp for mutants F and G, respectively. These data also included the DNA sequence of plasmid pZMR100 (5,580 nucleotides).

### Electron microscopy.

B. bacteriovorus cells and phage preparations were visualized using transmission electron microscopy. 15 μl of sample was placed on a carbon Formvar grid (Agar Scientific) for 5 min before being removed, and 15 μl of 0.5% uranyl acetate was added for 1 min before the grid was dried. Samples were imaged using a JEOL JEM1010 electron microscope.

### Predation kinetics experiments.

Predation kinetics were assayed as described and explained in Results: experimental measurements were taken in triplicate, and viable counting was used to enumerate phage, B. bacteriovorus, and E. coli. Two separate biological repeats of the experiment were run over 48 h, with enumerations of all three populations every 2 h by a team of four people.

The starting prey cultures had to be established by experimentation to produce prey cells that were suitable for both B. bacteriovorus and phage predation. In the lab, B. bacteriovorus predation is usually studied using stationary-phase prey, whereas phage predation typically requires exponentially growing prey; here, our setup resulted in late-log-phase prey cells that were preyed upon by both predators. E. coli S17-1 prey cells were pregrown for 16 h shaken at 37°C in YT broth. They were added, still in the YT broth, to 100 ml of calcium HEPES buffer (2 mM CaCl_2_, 25 mM HEPES [pH 7.8]) to give a final OD_600_ of 0.75 U (typically 20 ml of overnight culture added to 100 ml of buffer), resulting in an average starting E. coli prey population in the experimental cultures of 2.9 × 10^8^ CFU/ml.

Into 100 ml of this prey suspension, 2 ml of an attack-phase culture of B. bacteriovorus HD100 was added (or 2 ml of calcium HEPES buffer to B. bacteriovorus-free controls), giving an average starting B. bacteriovorus count in the experimental cultures of 2.8 × 10^6^ PFU/ml. To this, 20 μl of a pure preparation of the halo phage was added, giving an average starting count in the experimental cultures of 3.7 × 10^6^ PFU/ml. Cultures were incubated at 29°C with shaking at 200 rpm, and samples were taken every 2 h.

At each time point, the OD_600_ was measured, and samples were plated onto the appropriate agar plates for the enumeration of E. coli (YT), bacteriophage halo (YPSC with kanamycin at 50 μg/ml, with S17-1 pZMR100 prey), and B. bacteriovorus HD100 (YPSC with phage-resistant S17-1 as prey).

### Mathematical modeling.

A family of ordinary differential equation (ODE) models were developed to describe the population dynamics. ODEs were ideal since the experimental data are at the population rather than the individual level and the ODE model can be solved rapidly (this is important since we had to simulate the model millions of times for the model selection and parameter inference). Figure 3 visualizes the variables, their interactions, and the equations of the base model with one prey type. Figure 4 does the same for the final model, as well as explaining the different model variants. Parameters are defined in Table S1 in the supplemental material. The full sets of equations and details on the ODE solver are given in the supplemental material. Each model variant was fitted to the experimental data shown in Fig. 2. A Bayesian framework for model selection and parameter inference was used to obtain estimates of the uncertainty of the model and parameters. As explicit likelihood functions cannot be derived, an approximate Bayesian computation (ABC) with a sequential Monte Carlo (ABC-SMC) algorithm was used as described by Stumpf and coworkers (55); for details of the procedure, see the supplemental material. Figure S3 and S4 show how the fit improves with decreasing tolerance, and Fig. S5 shows how the accepted parameter ranges narrow down increasingly from the broad priors. The objective choice of typical parameter sets via PCA is shown in Fig. S7. The open source code for running the simulations and the model selection and fitting are available as supplemental code.

### Data availability.

The nucleotide sequences derived in this work have been deposited with GenBank: the bacteriophage halo partial genome sequence under accession number GQ495225.1 (https://www.ncbi.nlm.nih.gov/nuccore/GQ495225) and the B. bacteriovorus angelus full-length 16S rRNA sequence under accession number GQ427200.1 (https://www.ncbi.nlm.nih.gov/nuccore/GQ427200.1/). The E. coli wild-type strain S17-1 genome sequence was deposited under accession number CP040667.1 (https://www.ncbi.nlm.nih.gov/nuccore/NZ_CP040667.1).

## Supplementary Material

Supplemental file 1
