# Supplementary material for: Dual Predation by Bacteriophage and Bdellovibrio bacteriovorus Can Eradicate Escherichia coli Prey in Situations where Single Predation Cannot
Source: J Bacteriol. 2020 Feb 25;202(6):e00629-19. doi: 10.1128/JB.00629-19 (PMC7043672; doi:10.1128/JB.00629-19)
Supplement: Supplemental file 1 [file JB.00629-19-s0001.pdf]

Dual Predation by Bacteriophage and *Bdellovibrio* Can Eradicate *E. coli* Prey in Situations Where Single Predation Cannot

Laura Hobley,[a*] J. Kimberley Summers,[b] Rob Till,[a] David S. Milner,[a*] Robert J. Atterbury,[a*] Amy Stroud,[a] Michael J. Capeness,[a*] Stephanie Gray,[a] Andreas Leidenroth,[a] Carey Lambert,[a] Ian Connerton,[c] Jamie Twycross,[d] Michelle Baker,[a*] Jess Tyson,[a] Jan-Ulrich Kreft,[b#] R. Elizabeth Sockett[a#]

Supplemental Text: Full description of the mathematical modelling process

Introduction to the models. We model a batch culture with all nutrients combined into a single abiotic resource (M for medium), a single prey species with up to four phenotypes (N_S, N_R, N_P, N_D; defined below), all of which display Monod growth kinetics, and two predators, *B. bacteriovorus* (P for predator) and bacteriophage (V for virus). The rate of predation by either predator is described either by a non-saturating (linear) or by a saturating functional response (Holling type II) (Fig. 4E). The *B. bacteriovorus* life cycle consists of two phases, an attack phase to search for and attach to prey and a bdelloplast phase during which the *B. bacteriovorus* cell is within the periplasm of a prey cell, modelled here as a distinct phase in the predation cycle (B for bdelloplast). *B. bacteriovorus* is known to have an unusually high mortality rate for bacteria so we include a mortality term (described below). The predation by the bacteriophage halo is also separated into two distinct phases, one represented again by either a non-saturating or a saturating functional response, and the second by the infected cell phase (I for infected) where the bacteriophage is replicating within the prey cell. Initial assumptions (that were later tested as described below) included that *B. bacteriovorus* predation followed saturating kinetics, whereas bacteriophage halo predation kinetics were non-saturating. During the bdelloplast (B) or infected prey (I) states, the host prey cell does not grow or replicate (and will not contribute to the viable prey count when comparing with experimental data) and the predator within the prey cell does not predate further until after lysis of the prey cell. In the experiments, there is regrowth of prey during halo phage predation that exceeds the growth in the absence of predators (Fig. 2A and B), therefore nutrients must have been released during predation as the amount of spent medium added with the prey cells into the Calcium HEPES buffer at the start of the experiments would not be sufficient for this amount of growth. This source of nutrients is assumed to derive from the lysis of the prey cells after predation by either the *B. bacteriovorus* or the bacteriophage. Fig. 4A shows the populations and interactions included in the final model and the corresponding equations are shown in Fig. 4B. The parameters, priors and fitted values are given in Table S1.

Numerical integration of the model. All model variants were implemented in MATLAB 8.6.0.267246 (R2015b) and solved using the ordinary differential equation solver ode45, which implements an explicit Runge-Kutta method.

Model fitting methodology. We used a Sequential Monte Carlo (SMC) method (1) to select model variants and fit corresponding parameter sets using increasingly strict selection criteria (tolerance levels), see Fig. S3. As the priors were uniform, and the perturbation kernels were symmetric, all parameter sets carried equal weight. Each competition between model variants was performed for multiple generations. For each

generation, 1,000 'particles' (models with associated parameter sets) were selected and models competed for up to 12 generations, while the thresholds for the acceptable distance between observed and simulated data were reduced from generation to generation.

Each parameter set was evaluated for its fit to the experimental data using the distance function:

$$average\ distance = \frac{\sqrt[2]{sum[(point\ distance)^2]}}{number\ of\ data\ points}$$

where for each pair of data points (experimental and simulated) the point distance was calculated as:

$$point\ distance = \big(log(simulation\ datum) - log(experimental\ datum)\big)$$

As the tolerance limit decreased from generation to generation, the distribution of distances for accepted fits became increasingly narrow, indicating that the model parameter set combinations were approaching the best fit possible for the model variants and parameter ranges being tested (Fig. S3).

We repeated the model selection and fitting process ten times to test for the reproducibility of its outcomes (Fig. S6).

Top level model variants (Fig. 4C) – prey types. In our hierarchical model selection process, the top level model variants differ in the number of prey types. All mid-level variants deal with different ways of converting between prey types and therefore depend on the number of prey types, which is why the process is hierarchical. We identified the number of prey types (1-3) required to explain the experimental results. N1 is the base model with 1 prey phenotype, the prey type sensitive to both predators (N_S), and its equations are:

$$\frac{dM}{dt} = -N_S \frac{\mu_N\, M}{(K_{M,N} + M) Y_{N/M}} + Y_{M/P}\, k_p\, B + Y_{M/V}\, k_v\, I \qquad \text{(N1a)}$$

$$\frac{dN_S}{dt} = N_S \frac{\mu_N\, M}{K_{M,N} + M} - P \frac{\mu_P\, N_S}{(K_{N,P} + N_S) Y_{B/N}} - V \frac{\mu_V\, N_S}{Y_{I/N}} \qquad \text{(N1b)}$$

$$\frac{dP}{dt} = k_P\, B - m\, P - P \frac{\mu_P\, N_S}{(K_{N,P} + N_S) Y_{B/P}} \qquad \text{(N1c)}$$

$$\frac{dB}{dt} = P \frac{\mu_P\, N_S}{K_{N,P} + N_S} - \frac{k_P\, B}{Y_{P/B}} \qquad \text{(N1d)}$$

$$\frac{dV}{dt} = k_V\, I - V \frac{\mu_V\, N_S}{Y_{I/V}} \qquad \text{(N1e)}$$

$$\frac{dI}{dt} = V \mu_V\, N_S - \frac{k_V\, I}{Y_{V/I}} \qquad \text{(N1f)}$$

Model N2 includes two prey phenotypes, the prey type sensitive to both predators (N_S) as in model N1 and one resistant to bacteriophage predation (but still sensitive to *B. bacteriovorus* predation; N_R). In this model variant, it is assumed that the bacteriophage resistant subpopulation was a small fraction present at the beginning of the experiment (given by the parameter F_R) and that phage resistance can arise in sensitive cells (at a rate of *de novo* mutations that is fitted). It is also assumed that *B. bacteriovorus* predation does not distinguish between these prey types, and that there is no effect on growth kinetics due to bacteriophage resistance (cf. our experimental results showing that phage resistance is due to a mutation in the ferric hydroxamate uptake gene *fhuA* that had no growth defect in the iron sufficient conditions of the experiment).

The equations for model N2 are:

$$\frac{dM}{dt} = -(N_S + N_R)\frac{\mu_N M}{(K_{M,N} + M)Y_{N/M}} + Y_{M/P} k_p B + Y_{M/V} k_v I \tag{N2a}$$

$$\frac{dN_S}{dt} = N_S \frac{\mu_N M}{K_{M,N} + M} - P\frac{\mu_P N_S}{(K_{N,P} + N_S + N_R)Y_{B/N}} - V\frac{\mu_V N_S}{Y_{I/N}} - k_M N_s \tag{N2b}$$

$$\frac{dN_R}{dt} = N_R \frac{\mu_N M}{K_{M,N}+M} - P\frac{\mu_P N_R}{(K_{N,P} + N_S + N_R)Y_{B/N}} + k_M N_s, N_R(t=0) = F_R N_S(t=0) \tag{N2c}$$

$$\frac{dP}{dt} = k_P B - m P - P\frac{\mu_P (N_S + N_R)}{(K_{N,P} + N_S + N_R)Y_{B/P}} \tag{N2d}$$

$$\frac{dB}{dt} = P\frac{\mu_P (N_S + N_R)}{K_{N,P} + N_S + N_R} - \frac{k_P B}{Y_{P/B}} \tag{N2e}$$

$$\frac{dV}{dt} = k_V I - V\frac{\mu_V N_S}{Y_{I/V}} \tag{N2f}$$

$$\frac{dI}{dt} = V \mu_V N_S - \frac{k_V I}{Y_{V/I}} \tag{N2g}$$

The third variant, N3, is model N2 with a third prey population that exhibits 'plastic' phenotypic resistance to *B. bacteriovorus* predation (N_P). Subsequent model levels consider the mechanisms by which this plastic resistance arises and by which conversion back to sensitive prey may occur. At this level, plastic resistance is modelled to arise intrinsically (spontaneously) at a fixed rate whilst reversion to sensitive is coupled to growth of the plastic resistant type. The equations for model variant N3 with these specifics are shown further below (equation set N3-IG).

The final variant, N4, is variant N3 with a fourth prey population with double resistance (N_D), i.e., heritable phage resistance and plastic phenotypic resistance to *B. bacteriovorus*.

Model variants with only sensitive prey (N1) or sensitive and phage resistant prey (N2) were not at all supported by the data. Model variant N3 was best supported and model variant N4 was selected with a frequency of ~1/3 (Fig. 5A), strongly supporting the existence of the three prey phenotypes: sensitive, phage resistant, and plastic resistant to *B. bacteriovorus*. The double resistant prey can exist as the two resistance mechanisms are independent, but predicted abundances are very low so in a natural environment with additional causes of mortality, they will be unlikely to survive.

Mid-level model variants (Fig. 4Di) – generating plastic resistance of prey to *B. bacteriovorus* predation. We then inferred by which mechanism plastic prey resistance to *B. bacteriovorus* predation arises, and how these plastic resistant prey revert to sensitive prey.

One variant assumes that conversion is intrinsic (spontaneous, without external trigger) in both directions, from sensitive to resistant and back. This is model N3-I (I for intrinsic conversion). Conversion occurs with a certain specific rate (k_D) and is proportional to prey cell density; likewise for reversion to sensitive (k_R). The growth rates of sensitive and plastic resistant prey are allowed to differ by the factor η_P, which is fitted and can be greater or less than 1 (Table S1).

The equations for variant N3-I are:

$$\frac{dM}{dt} = -(N_S + N_R + N_P\eta_P)\frac{\mu_N\, M}{(K_{M,N} + M)Y_{N/M}} + Y_{M/P}k_p\, B + Y_{M/V}\, k_v\, I \qquad \text{(N3-Ia)}$$

$$\frac{dN_S}{dt} = N_S\frac{\mu_N\, M}{K_{M,N} + M} - P\frac{\mu_P\, N_S}{(K_{N,P} + N_S + N_P + N_R)Y_{B/N}} - V\frac{\mu_V\, N_S}{Y_{I/N}} - \boldsymbol{k_D\, N_S} + \boldsymbol{k_R\, N_P} - k_M\, N_s \qquad \text{(N3-Ib)}$$

$$\frac{dN_P}{dt} = N_P\frac{\eta_P\mu_N\, M}{K_{M,N} + M} - V\frac{\mu_V\, N_P}{Y_{I/N}} + \boldsymbol{k_D\, N_S} - \boldsymbol{k_R\, N_P} \qquad \text{(N3-Ic)}$$

$$\frac{dN_R}{dt} = N_R\frac{\mu_N\, M}{K_{M,N} + M} - P\frac{\mu_P\, N_R}{(K_{N,P} + N_S + N_P + N_R)Y_{B/N}} + k_M\, N_s\,, N_R(t=0) = F_R\, N_S(t=0) \qquad \text{(N3-Id)}$$

$$\frac{dP}{dt} = k_P\, B - m\, P - P\frac{\mu_P(N_S + N_R)}{(K_{N,P} + N_S + N_P + N_R)Y_{B/P}} \qquad \text{(N3-Ie)}$$

$$\frac{dB}{dt} = P\frac{\mu_P(N_S + N_R)}{K_{N,P} + N_S + N_P + N_R} - \frac{k_P\, B}{Y_{P/B}} \qquad \text{(N3-If)}$$

$$\frac{dV}{dt} = k_V\, I - V\frac{\mu_V(N_S + N_P)}{Y_{I/V}} \qquad \text{(N3-Ig)}$$

$$\frac{dI}{dt} = V\,\mu_V(N_S + N_P) - \frac{k_V\, I}{Y_{V/I}} \qquad \text{(N3-Ih)}$$

Another variant also assumes that the plastic resistance occurs spontaneously, but differs in that these plastic resistant prey revert to sensitive in a growth-coupled manner, model N3-IG (IG for intrinsic conversion with growth-coupled back conversion). This is the sub-model of N3 used for the top-level model selection and given by these equations:

$$\frac{dM}{dt} = -(N_S + N_R + N_P)\frac{\mu_N\, M}{(K_{M,N} + M)Y_{N/M}} + Y_{M/P}\, k_p\, B + Y_{M/V}\, k_v\, I \qquad \text{(N3-IGa)}$$

$$\frac{dN_S}{dt} = (N_S + \boldsymbol{N_P})\frac{\mu_N\, M}{K_{M,N} + M} - P\frac{\mu_P\, N_S}{(K_{N,P} + N_S + N_P + N_R)Y_{B/N}} - V\,\frac{\mu_V\, N_S}{Y_{I/N}} - \boldsymbol{k_D\, N_S} - k_M\, N_s \qquad \text{(N3-IGb)}$$

$$\frac{dN_P}{dt} = -V\,\frac{\mu_V\, N_P}{Y_{I/N}} + \boldsymbol{k_D\, N_S} \qquad \text{(N3-IGc)}$$

$$\frac{dN_R}{dt} = N_R\frac{\mu_N\, M}{K_{M,N} + M} - P\frac{\mu_P\, N_R}{(K_{N,P} + N_S + N_P + N_R)Y_{B/N}} + k_M\, N_s\,, N_R(t=0) = F_R\, N_S(t=0) \qquad \text{(N3-IGd)}$$

$$\frac{dP}{dt} = k_P\, B - m\, P - P\frac{\mu_P(N_S + N_R)}{(K_{N,P} + N_S + N_P + N_R)Y_{B/P}} \qquad \text{(N3-IGe)}$$

$$\frac{dB}{dt} = P\frac{\mu_P(N_S + N_R)}{K_{N,P} + N_S + N_P + N_R} - \frac{k_P\, B}{Y_{P/B}} \qquad \text{(N3-IGf)}$$

$$\frac{dV}{dt} = k_V\, I - V\,\frac{\mu_V(N_S + N_P)}{Y_{I/V}} \qquad \text{(N3-IGg)}$$

$$\frac{dI}{dt} = V\,\mu_V(N_S + N_P) - \frac{k_V\, I}{Y_{V/I}} \qquad \text{(N3-IGh)}$$

A third variant assumes that plastic resistance arises due to the production of some lumped signal S, resulting from predation. Such signals could be released during the lysis of prey, so this model includes terms for a signal being released from both the lysis of bdelloplasts and the lysis of phage-infected prey. N3-S models back conversion of plastic resistant prey to sensitive as being intrinsic, as in variant N3-I above. The equations for model variant N3-S are:

$$\frac{dM}{dt} = -(N_S + N_R + N_P\eta_P)\frac{\mu_N\, M}{(K_{M,N} + M)Y_{N/M}} + Y_{M/P}k_p\, B + Y_{M/V}\, k_v\, I \qquad \text{(N3-Sa)}$$

$$\frac{dN_S}{dt} = N_S\frac{\mu_N\, M}{K_{M,N} + M} - P\frac{\mu_P\, N_S}{(K_{N,P} + N_S + N_P + N_R)Y_{B/N}} - V\frac{\mu_V\, N_S}{Y_{I/N}} - \boldsymbol{k_D\, S\, N_S} + \boldsymbol{k_R\, N_P} - k_M\, N_s \qquad \text{(N3-Sb)}$$

$$\frac{dN_P}{dt} = N_P\frac{\eta_P\, \mu_N\, M}{K_{M,N} + M} - V\frac{\mu_V\, N_P}{Y_{I/N}} + \boldsymbol{k_D\, S\, N_S} - \boldsymbol{k_R\, N_P} \qquad \text{(N3-Sc)}$$

$$\frac{dN_R}{dt} = N_R\frac{\mu_N\, M}{K_{M,N} + M} - P\frac{\mu_P\, N_R}{(K_{N,P} + N_S + N_P + N_R)Y_{B/N}} + k_M\, N_s\, , N_R(t=0) = F_R\, N_S(t=0) \qquad \text{(N3-Sd)}$$

$$\frac{dP}{dt} = k_P\, B - m\, P - P\frac{\mu_P(N_S + N_R)}{(K_{N,P} + N_S + N_P + N_R)Y_{B/P}} \qquad \text{(N3-Se)}$$

$$\frac{dB}{dt} = P\frac{\mu_P(N_S + N_R)}{K_{N,P} + N_S + N_P + N_R} - \frac{k_P\, B}{Y_{P/B}} \qquad \text{(N3-Sf)}$$

$$\frac{dV}{dt} = k_V\, I - V\frac{\mu_V(N_S + N_P)}{Y_{I/V}} \qquad \text{(N3-Sg)}$$

$$\frac{dI}{dt} = V\mu_V(N_S + N_P) - \frac{k_V\, I}{Y_{V/I}} \qquad \text{(N3-Sh)}$$

$$\boldsymbol{\frac{dS}{dt} = \frac{k_P\, B}{Y_{P/B}} + \frac{k_V\, I}{Y_{V/I}}} \qquad \text{(N3-Si)}$$

Competing these three variants (Fig. 5B and S6) revealed that the data gave very little support for model N3-I, some support (between 9.4% and 27% of accepted parameter sets) for variant N3-S, and stronger support for variant N3-IG (between 62% to 89% of accepted parameter sets).

We then decided to try a combination of the two variants that were supported by the data, where the plastic resistant prey arise in response to a predation signal and the back conversion to sensitive is coupled to growth, giving model N3-SG:

$$\frac{dM}{dt} = -(N_S + N_R + N_P)\frac{\mu_N\, M}{(K_{M,N} + M)Y_{N/M}} + Y_{M/P}\, k_p\, B + Y_{M/V}\, k_v\, I \qquad \text{(N3-SGa)}$$

$$\frac{dN_S}{dt} = (N_S + \boldsymbol{N_P})\frac{\mu_N\, M}{K_{M,N} + M} - P\frac{\mu_P\, N_S}{(K_{N,P} + N_S + N_P + N_R)Y_{B/N}} - V\frac{\mu_V\, N_S}{Y_{I/N}} - \boldsymbol{k_D\, S\, N_S} - k_M\, N_s \qquad \text{(N3-SGb)}$$

$$\frac{dN_P}{dt} = -V\frac{\mu_V\, N_P}{Y_{I/N}} + \boldsymbol{k_D\, S\, N_S} \qquad \text{(N3-SGc)}$$

$$\frac{dN_R}{dt} = N_R\frac{\mu_N\, M}{K_{M,N} + M} - P\frac{\mu_P\, N_R}{(K_{N,P} + N_S + N_P + N_R)Y_{B/N}} + k_M\, N_s\,, N_R(t=0) = F_R\, N_S(t=0) \qquad \text{(N3-SGd)}$$

$$\frac{dP}{dt} = k_P\, B - m\, P - P\frac{\mu_P(N_S + N_R)}{(K_{N,P} + N_S + N_P + N_R)Y_{B/P}} \qquad \text{(N3-SGe)}$$

$$\frac{dB}{dt} = P\frac{\mu_P(N_S + N_R)}{K_{N,P} + N_S + N_P + N_R} - \frac{k_P\, B}{Y_{P/B}} \qquad \text{(N3-SGf)}$$

$$\frac{dV}{dt} = k_V\, I - V\frac{\mu_V(N_S + N_P)}{Y_{I/V}} \qquad \text{(N3-SGg)}$$

$$\frac{dI}{dt} = V\,\mu_V(N_S + N_P) - \frac{k_V\, I}{Y_{V/I}} \qquad \text{(N3-SGh)}$$

$$\boldsymbol{\frac{dS}{dt} = \frac{k_P\, B}{Y_{P/B}} + \frac{k_V\, I}{Y_{V/I}}} \qquad \text{(N3-SGi)}$$

Competing variants N3-S, N3-IG and N3-SG showed that the data supported the (re)combined variant N3-SG much better than its parents N3-S and N3-IG (Fig. 5C and S6). Thus, we can infer from the modelling that plastic resistance arises as a result of some signal(s) released upon lysis of infected prey, and that the growth of plastic resistant prey results in the production of sensitive prey.

Mid level model variants (Fig. 4Di) – source of the signal resulting in plastic resistance. We next inferred potential sources for the production of the signal triggering conversion to plastic resistance. Model N3-SG assumes that the signal is generated by lysis of both *B. bacteriovorus* infected prey (bdelloplasts) and bacteriophage infected prey. However, the signal may be specifically released by lysis of B or I. Variant N3-S**V**G assumes signal production from lysis of the virus (bacteriophage) infected prey I. This is the same as variant N3-SG except that equation N3-SGi is replaced by equation N3-S**V**Gi:

$$\frac{dS}{dt} = \frac{k_V\, I}{Y_{V/I}} \qquad \text{(N3-S\textbf{V}Gi)}$$

The alternative variant is that the signal was specifically released by lysis of B, the bdelloplasts, giving model N3-S**B**G. Here, the equations are again the same as variant N3-SG except that equation N3-SGi replaced by N3-S**B**Gi:

$$\frac{dS}{dt} = \frac{k_P\, B}{Y_{P/B}} \qquad \text{(N3-S\textbf{B}Gi)}$$

Competing these three variants (Fig. 5D and S6) showed no support for a model where the signal is generated by lysis of the bacteriophage-infected prey only. The support for the other two variants was almost equal. Since there was no evidence for phage involvement in signal generation, the model N3-S**B**G where all signal is derived from *B. bacteriovorus* predation is the logical choice.

Mid level model variants (Fig. 4Dii) – generating phage resistant prey. The successful model variant N3 (or more precisely N3-IG) was then used as a basis to infer how phage resistance could arise. One variant assumed that all prey were sensitive to phage at the outset of the experiment but that the resistant population N_R could develop by *de novo* mutations during the experiment with mutation rate k_M (Table S1), giving model N3-RD (from <u>r</u>esistance <u>d</u>eveloping):

$$\frac{dM}{dt} = -(N_S + N_R + N_P)\frac{\mu_N\, M}{(K_{M,N} + M)Y_{N/M}} + Y_{M/P} k_p\, B + Y_{M/V}\, k_v\, I \qquad \text{(N3-RDa)}$$

$$\frac{dN_S}{dt} = (N_S + N_P)\frac{\mu_N\, M}{K_{M,N} + M} - P\frac{\mu_P\, N_S}{(K_{N,P} + N_S + N_P + N_R)Y_{B/N}} - V\frac{\mu_V\, N_S}{Y_{I/N}} - k_D\, N_S - \boldsymbol{k_M\, N_S} \qquad \text{(N3-RDb)}$$

$$\frac{dN_P}{dt} = -V\frac{\mu_V\, N_P}{Y_{I/N}} + k_D\, N_S \qquad \text{(N3-RDc)}$$

$$\frac{dN_R}{dt} = N_R\frac{\mu_N\, M}{K_{M,N} + M} - P\frac{\mu_P\, N_R}{(K_{N,P} + N_S + N_P + N_R)Y_{B/N}} + \boldsymbol{k_M\, N_S, N_R(t=0) = 0} \qquad \text{(N3-RDd)}$$

$$\frac{dP}{dt} = k_P\, B - m\, P - P\frac{\mu_P(N_S + N_R)}{(K_{N,P} + N_S + N_P + N_R)Y_{B/P}} \qquad \text{(N3-RDe)}$$

$$\frac{dB}{dt} = P\frac{\mu_P(N_S + N_R)}{K_{N,P} + N_S + N_P + N_R} - \frac{k_P\, B}{Y_{P/B}} \qquad \text{(N3-RDf)}$$

$$\frac{dV}{dt} = k_V\, I - V\frac{\mu_V(N_S + N_P)}{Y_{I/V}} \qquad \text{(N3-RDg)}$$

$$\frac{dI}{dt} = V\,\mu_V(N_S + N_P) - \frac{k_V\, I}{Y_{V/I}} \qquad \text{(N3-RDh)}$$

Another variant assumed that a fraction F_R (Table S1) of the prey population was already <u>r</u>esistant <u>i</u>nitially, but that further resistance via mutation did not develop (model N3-RI):

$$\frac{dM}{dt} = -(N_S + N_R + N_P)\frac{\mu_N\, M}{(K_{M,N} + M)Y_{N/M}} + Y_{M/P} k_p\, B + Y_{M/V}\, k_v\, I \qquad \text{(N3-RIa)}$$

$$\frac{dN_S}{dt} = (N_S + N_P)\frac{\mu_N\, M}{K_{M,N} + M} - P\frac{\mu_P\, N_S}{(K_{N,P} + N_S + N_P + N_R)Y_{B/N}} - V\frac{\mu_V\, N_S}{Y_{I/N}} - k_D\, N_S \qquad \text{(N3-RIb)}$$

$$\frac{dN_P}{dt} = -V\frac{\mu_V\, N_P}{Y_{I/N}} + k_D\, N_S \qquad \text{(N3-RIc)}$$

$$\frac{dN_R}{dt} = N_R\frac{\mu_N\, M}{K_{M,N} + M} - P\frac{\mu_P\, N_R}{(K_{N,P} + N_S + N_P + N_R)Y_{B/N}}, \boldsymbol{N_R(t=0) = F_R\, N_S(t=0)} \qquad \text{(N3-RId)}$$

$$\frac{dP}{dt} = k_P\, B - m\, P - P\frac{\mu_P(N_S + N_R)}{(K_{N,P} + N_S + N_P + N_R)Y_{B/P}} \qquad \text{(N3-RIe)}$$

$$\frac{dB}{dt} = P\frac{\mu_P(N_S + N_R)}{K_{N,P} + N_S + N_P + N_R} - \frac{k_P\, B}{Y_{P/B}} \qquad \text{(N3-RIf)}$$

$$\frac{dV}{dt} = k_V\, I - V\frac{\mu_V(N_S + N_P)}{Y_{I/V}} \qquad \text{(N3-RIg)}$$

$$\frac{dI}{dt} = V\,\mu_V(N_S + N_P) - \frac{k_V\, I}{Y_{V/I}} \qquad \text{(N3-RIh)}$$

The combined variant N3-RID where phage resistance is both present as an initial subpopulation of the prey and also arises due to *de novo* mutations during the course of the experiment was better supported by the data than N3-RI; N3-RD was not supported at all (Fig. 5E). Model N3-RID is identical to the original model variant N3 described above.

Low level model variants – saturating or non-saturating predation kinetics (Fig. 4E). The model was initially constructed assuming that bacteriophage predation would not saturate with increasing prey density but that *B. bacteriovorus* predation would saturate (Holling type II kinetics) due to its longer handling time (time for attachment and prey entry). This assumption was challenged by testing all four combinations of saturating and non-saturating functional responses for the two predators (Fig. 5F). The previously used model variant N3-SBG (see above) was in fact N3-SBG-Pii-Vi where Pii means Predator follows saturating kinetics and Vi means that the Virus follows non-saturating or type I kinetics. This original model was competed against variants N3-SBG-Pi-Vi (both predators following non-saturating kinetics), N3-SBG-Pi-Vii (*B. bacteriovorus* following non-saturating and phage saturating kinetics) and N3-SBG-Vii-Pii (both predators following saturating kinetics). The previously used variant N3-SBG-Pii-Vi consistently outcompeted all other variants (Fig. 5F).

Low level model variants – fixed or fitted *B. bacteriovorus* mortality. There remained some discrepancy in the best fitting model with regards to the decline in *B. bacteriovorus* numbers seen in the experimental data. Therefore, we allowed *B. bacteriovorus* mortality, which had previously been fixed at 0.06 h^{-1}, a value taken from (2), to be fitted as an additional parameter. The N3-SBG-Pii-Vi model with fitted *B. bacteriovorus* mortality clearly outcompeted the variant with fixed mortality (Fig. 5G).

The final model. The variant with fitted mortality became the final model, N3-SBG-RID-Pii-Vi-fitted-mortality or N3-SBG for short, which was used in the simulations of the experimental data shown in Fig. 6. Fitted parameters are given in Table S1. The equations are shown in Fig. 4B and are the same as N3-SBG above.

Picking typical parameters with PCA (Fig. S7). In order to objectively pick a typical parameter set to be used in model simulations shown in Fig. 6 from the many accepted fits, we used Principal Component Analysis (PCA) to find the parameter set in the centre of the cloud of points, which we regard as 'typical' or 'average'. We applied this PCA idea to the parameter sets obtained by fitting the final model to all the experimental data (fitting 15 parameters, one (*B. bacteriovorus* burst size) was fixed). One of these parameter sets was chosen (Fig. S7). The values are given in Table S1 and the fit to the experimental data is shown in Fig. 6A-D. We did the same for the parameter sets fitted to all data without dual predation, the resulting typical parameter set is also given in Table S1 and the fit to experimental data is shown in Fig. 6E-H. The two fits, to all data or to all data without dual predation, are very similar, suggesting that the combined action of the two predators does not require any additional terms for direct interactions between the two, and that the observed kinetics of dual predation can be predicted from the actions of each predator alone.

CODE for Modelling:
link at https://github.com/kreft/predatorprey

References

1. Toni T, Welch D, Strelkowa N, Ipsen A, Stumpf MP. 2009. Approximate Bayesian computation scheme for parameter inference and model selection in dynamical systems. J R Soc Interface 6:187-202.
2. Hespell RB, Rosson RA, Thomashow MF, Rittenberg SC. 1973. Respiration of Bdellovibrio bacteriovorus strain 109J and its energy substrates for intraperiplasmic growth. J Bacteriol 113:1280-8.

Supplemental Legends

Table S1. Description of model parameters with their symbols and units. The ranges used for parameter fitting (priors) and results of ABC-SMC fitting are also given. Typical fitted parameters were identified using PCA, see Supplemental text and Fig. S7 for an explanation.

Fig. S1. Isolation and peptide analysis of a bacteriophage halo protein from the 0.22µm filtrate. **(A)** SDS-page analysis of the purified bacteriophage halo showing multiple protein bands. The highlighted ~30 kDa band was extracted and analysed by MALDI QToF MS, showing homology to protein RTP27 of the rosette-tailed bacteriophage RTP. **(B)** Five peptides (bold) found by MALDI QToF MS analysis of the 30 kDa protein from panel (A) with homology to the 34 kDa protein RTP27 (GenBank accession no: CAJ42231.1, EMBL Accession Number AM156909.1) of a rosette-tailed phage (RTP) of *E. coli* (32).

Fig. S2. Plaque morphology from *E. coli* lawns on agar overlay plates of **(A)** purified *B. bacteriovorus* angelus alone after 6 days incubation and **(B)** when co-cultured with bacteriophage halo after 1 day of incubation, showing differences in plaque dimensions (bracketed on plate images taken at identical magnification), morphology and speed of plaque formation.

Fig. S3. The fit of the final model improves from generation to generation in the ABC-SMC method Each generation lowered the threshold for accepting a fit. In the final generation, all accepted fits have a similar distance between simulated and experimental data, showing that fits with further reduced distance did not occur. None of the accepted fits in the first generation would have been accepted in the last generation. The x axis has no meaning and is simply used to spread out the data. See Supplemental Text for the distance measure.

Fig. S4. Improved model fit. The fit of the final model to all data improves over the generations, showing a subset of the generations in Fig. S3.

Fig. S5. Convergence of fitted parameters from generation to generation, We started from the uniform priors to the values after the final generation 12. Parameters that are better informed by the experimental data reach a more narrow spread of fitted values. Medians and quartiles from the final generation are shown in Supplemental Table S1. Note the log scale for the parameters.

Fig. S6. Box plots showing the variation amongst the 10 repeats of model selection. We repeated the model selection procedure shown in Fig. 4B-D ten times because finding 1,000 acceptable parameter sets with a Monte Carlo method could lead to some variation between runs of the ABC-SMC algorithm and it is good to check whether these difference are large. Box plots show the variation amongst the 10 repeats. We only show repeats for sub-model selections where the outcome was not 100% in favour of one model variant. The Bayes factor is the ratio of the times model X was accepted versus model Y.

Fig. S7. Objective selection of a typical parameter set. Selection was from the hundreds of parameter sets that gave acceptable fits and were nearly equally good (see Fig. S3). Principal Component Analysis (PCA) was used to find the centre of the cloud of parameter sets in 15 dimensional parameter space. Four parameter sets that were closest to the centre were picked and used to run simulations. Results of these 4

simulations were indistinguishable by eye and one of these 4 parameter sets was then chosen as 'typical' and used in Fig. 5 and reported in Table S1.

Fig. S8. Effect of varying initial densities. The model was simulated using the typical parameter values fitted to all data in Table S1, with either initial values from the experimental data (solid lines), one order of magnitude higher than these values (dashed lines) or one order of magnitude lower (dotted lines). Blue: *E. coli* prey, Red: *B. bacteriovorus*, Green: bacteriophage halo, Pink: medium. **(A)** Effects of initial prey density on prey only scenario. **(B-C)** Effects of varying either initial prey **(B)** or initial *Bdellovibrio* **(C)** densities on *Bdellovibrio* only predation. **(D-E)** Effects of varying either initial prey **(D)** or initial halophage **(E)** densities on halophage only predation. **(F-H)** Effects of varying either initial prey **(F)**, initial *Bdellovibrio* **(G)** or initial halophage **(H)** densities on dual predation.

Table S1. Description of model parameters with their symbols and units. The ranges used for parameter fitting (priors) and results of ABC-SMC fitting are also given. Typical fitted parameters were identified using PCA, see Supplementary text S1 and Fig. S7 for an explanation.

Parameters	Units	Priors for fitting		Statistics of fitted parameters		Typical fitted parameters	
		Minimum value	Maximum value	Median	25th – 75th percentile	Using all data for fitting	Without using dual predator data
Initial phage resistant fraction of prey (F_R)	dimensionless	$1.0x10^{-6}$	$1.0x10^{-4}$	$3.6x10^{-6}$	$2.3x10^{-6}$<br>$5.4\ x10^{-6}$	$8.6x10^{-6}$	$3.9x10^{-6}$
Prey maximum growth rate (μ_N)	h^{-1}	¼ ln(2)	3 ln(2)	0.47	0.38<br>0.60	0.46	1.3
Prey affinity for medium ($K_{M,N}$)	pg ml^{-1}	$1.0x10^{7}$	$4.0x10^{10}$	$3.8x10^{7}$	$1.9x10^{7}$<br>$6.7x10^{7}$	$4.2x10^{7}$	$1.6x10^{8}$
Growth rate scaling for plastic resistant prey (η_P)	dimensionless	0.4	2	0.98	0.72<br>1.3	Parameter not in final model	
Yield of prey per medium ($Y_{N/M}$)	prey cells<br>pg $substrate^{-1}$	1	10	2.2	1.5<br>3.0	1.2	1.8
B. bacteriovorus maximum attack rate – non-saturating (μ_P)	bdelloplast cells<br>*B. bacteriovorus* $cells^{-1}$<br>prey $cells^{-1}$<br>h^{-1}	$2.1x10^{-9}$	$5.1x10^{-7}$	$2.3x10^{-8}$	$1.6x10^{-8}$<br>$3.0x10^{-8}$	Parameter not in final model	
B. bacteriovorus maximum attack rate – Holling type II (μ_P)	bdelloplast cells<br>*B. bacteriovorus* $cells^{-1}$<br>h^{-1}	$8.1x10^{-2}$	$2.1x10^{1}$	0.36	0.31<br>0.43	0.33	0.36
B. bacteriovorus affinity for prey ($K_{N,P}$)	prey cells ml^{-1}	$2.0x10^{4}$	$1.0x10^{9}$	$3.2x10^{6}$	$2.5x10^{6}$<br>$4.2x10^{6}$	$3.2x10^{6}$	$3.6x10^{6}$
Bdelloplast maturation rate (k_P)	*B. bacteriovorus* cells<br>bdelloplast $cells^{-1}$<br>h^{-1}	$7.0x10^{-1}$	$2.5x10^{0}$	0.86	0.78<br>0.98	1.1	1.1
Bacteriophage halo maximum attack rate – non-saturating (μ_V)	infected cells<br>bacteriophage $virions^{-1}$<br>prey $cells^{-1}$<br>h^{-1}	$1.0x10^{-10}$	$1.0x10^{-7}$	$1.6x10^{-9}$	$1.2x10^{-9}$<br>$2.2x10^{-9}$	$3.9x10^{-9}$	$1.9x10^{-9}$
Bacteriophage halo maximum attack rate – Holling type II (μ_V)	infected cells<br>bacteriophage $virions^{-1}$<br>h^{-1}	$3.2x10^{-3}$	$3.2x10^{0}$	0.43	0.22<br>0.91	Parameters not in final model	

Parameters	Units	Priors for fitting		Statistics of fitted parameters		Typical fitted parameters	
		Minimum value	Maximum value	Median	25th – 75th percentile	Using all data for fitting	Without using dual predator data
Bacteriophage halo affinity for prey ($K_{N,V}$)	prey cells ml^{-1}	$1.3x10^{5}$	$1.3x10^{10}$	$1.0x10^{8}$	$4.2x10^{8}$<br>$5.0x10^{8}$		
Infected cell lysis rate (k_V)	bacteriophage virions infected $cells^{-1}$ h^{-1}	$1.0x10^{0}$	$5.0x10^{2}$	12	8.1<br>18	4.2	8.7
Bacteriophage halo burst size ($Y_{V/I}$)	bacteriophage virions infected $cells^{-1}$	$5.0x10^{0}$	$2.0x10^{2}$	23	20<br>27	24	34
Nutrients released on *B. bacteriovorus* lysis ($Y_{M/P}$)	pg nutrients *B. bacteriovorus* $cell^{-1}$	$2.0x10^{-5}$	$2.0x10^{-1}$	$1.2x10^{-3}$	$2.7x10^{-4}$<br>$5.5x10^{-3}$	$3.3x10^{-3}$	$2.8x10^{-3}$
Nutrients released on phage lysis ($Y_{M/V}$)	pg nutrients bacteriophage $virion^{-1}$	$1.0x10^{-4}$	$3.2x10^{-1}$	$1.7x10^{-2}$	$8.8x10^{-3}$<br>$3.8x10^{-2}$	$2.1x10^{-2}$	$6.9x10^{-3}$
Rate of developing *B. bacteriovorus* plastic resistance – without signal (k_D)	h^{-1}	$5.0x10^{-8}$	$5.0x10^{-4}$	$5.0x10^{-5}$	$2.0x10^{-5}$<br>$1.1x10^{-4}$	Parameter not in final model	
Rate of developing *B. bacteriovorus* plastic resistance – with signal (k_D)	prey $cells^{-1}$ h^{-1}	$5.0x10^{-14}$	$5.0x10^{-10}$	$1.7x10^{-12}$	$9.4x10^{-13}$<br>$2.9x10^{-12}$	$4.0x10^{-12}$	$1.3x10^{-12}$
Rate of reversion to sensitive prey (k_R)	h^{-1}	$5.0x10^{-7}$	$5.0x10^{-3}$	$2.9x10^{-5}$	$6.4x10^{-5}$<br>$1.7x10^{-4}$	Parameter not in final model	
Rate of *de novo* phage resistance mutations (k_M)	h^{-1}	$1x10^{-10}$	$1x10^{-8}$	$7.0x10^{-10}$	$3.5x10^{-10}$<br>$1.6x10^{-9}$	$7.5 x 10^{-10}$	$4.9x10^{-9}$
Predator mortality rate (m)	h^{-1}	0.04	0.15	$6.1x10^{-2}$	$5.2x10^{-2}$<br>$7.2x10^{-2}$	$6.4x10^{-2}$	$9.5x10^{-2}$
B. bacteriovorus burst size ($Y_{P/B}$)	*B. bacteriovorus* cells $bdelloplast^{-1}$	Fixed to literature value of 4.17 from Fenton et al. (2010), reference 22 in main paper					

A

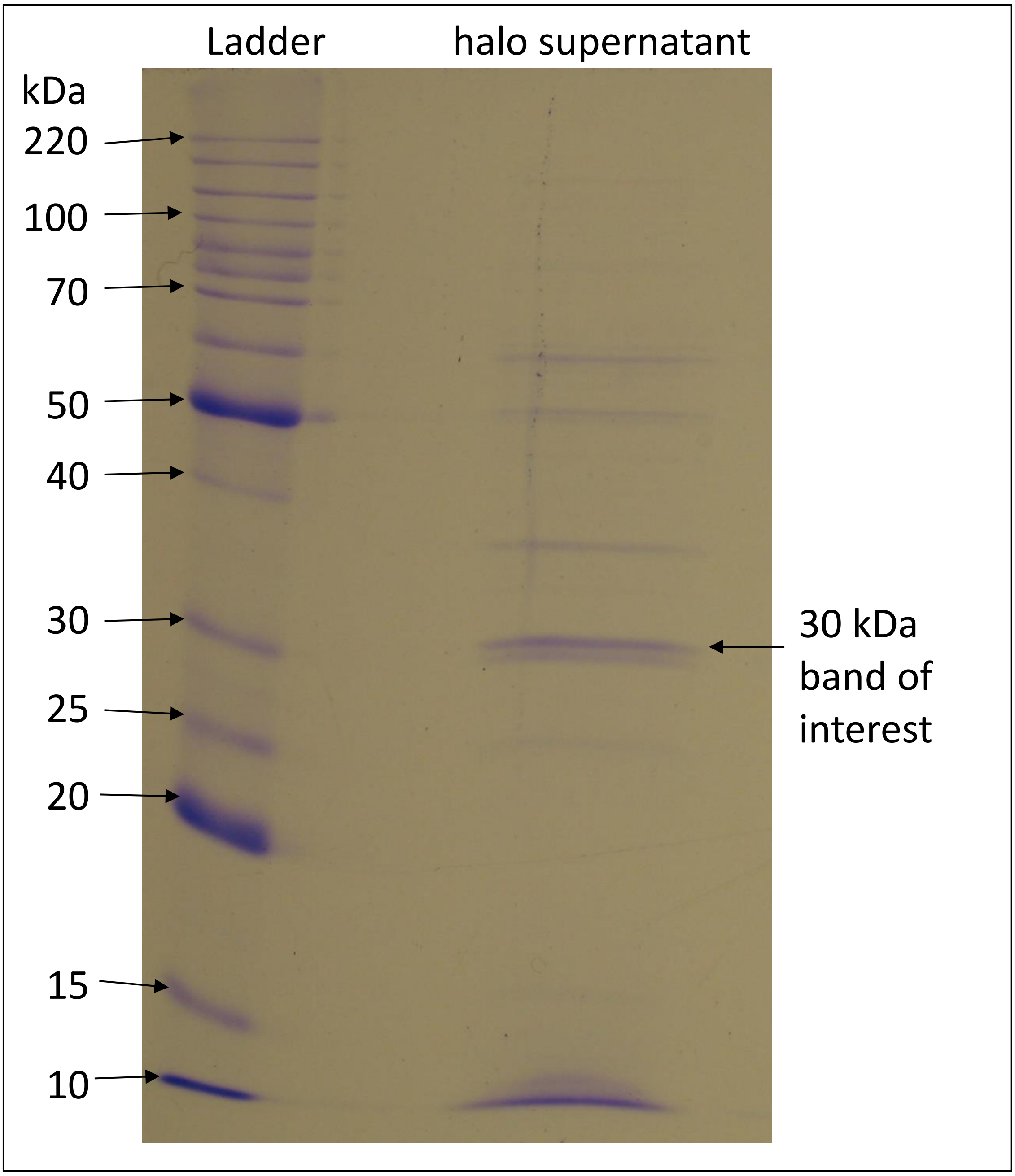

B

1 MAIKFDAEQA KITTHLEQMG VEKADAAGIW AVSQLTAALN RAYEKEYAEN
51 SVVNIFPVTN EIPGHAK**YFE YPEFDGVGIA QIIADYSDDL PLVDAFMTEK**
101 QGKVFR**FGNA FLISTDEIK**A GAATGQSLSA RK**QALAFEAH DNLLDK**LVWS
151 GSAPHGIVSV FDQPNINNVV ATPNWSVPQN AIDDVTAMID AVESSTQGLH
201 HVTDILLPAS ARR**VMQGLVP QTNLSYGELF TRNNPGLTIR FLQFLDNYDG**
251 **AGGK**AALAFE K**SPLNMSIEI PEVTNVLPAQ PK**DLHFRYPV TSKATGLIVY
301 RPLTMAVIKG ITFA

Fig. S1. Isolation and peptide analysis of a bacteriophage halo protein from the 0.22µm filtrate. **(A)** SDS-page analysis of the purified bacteriophage halo showing multiple protein bands. The highlighted ~30 kDa band was extracted and analysed by MALDI QToF MS, showing homology to protein RTP27 of the rosette-tailed bacteriophage RTP. **(B)** Five peptides (bold) found by MALDI QToF MS analysis of the 30 kDa protein from panel (A) with homology to the 34 kDa protein RTP27 (GenBank accession no: CAJ42231.1, EMBL Accession Number AM156909.1) of a rosette-tailed phage (RTP) of *E. coli* (32).

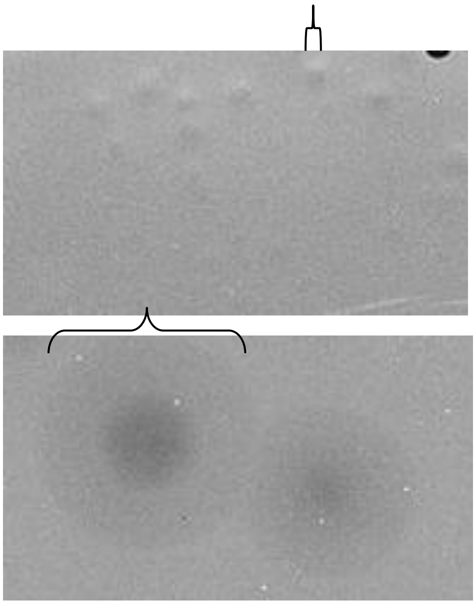

Fig. S2. Plaque morphology from *E. coli* lawns on agar overlay plates of **(A)** purified *B. bacteriovorus* angelus alone after 6 days incubation and **(B)** when co-cultured with bacteriophage halo after 1 day of incubation, showing differences in plaque dimensions (bracketed on plate images taken at identical magnification), morphology and speed of plaque formation.

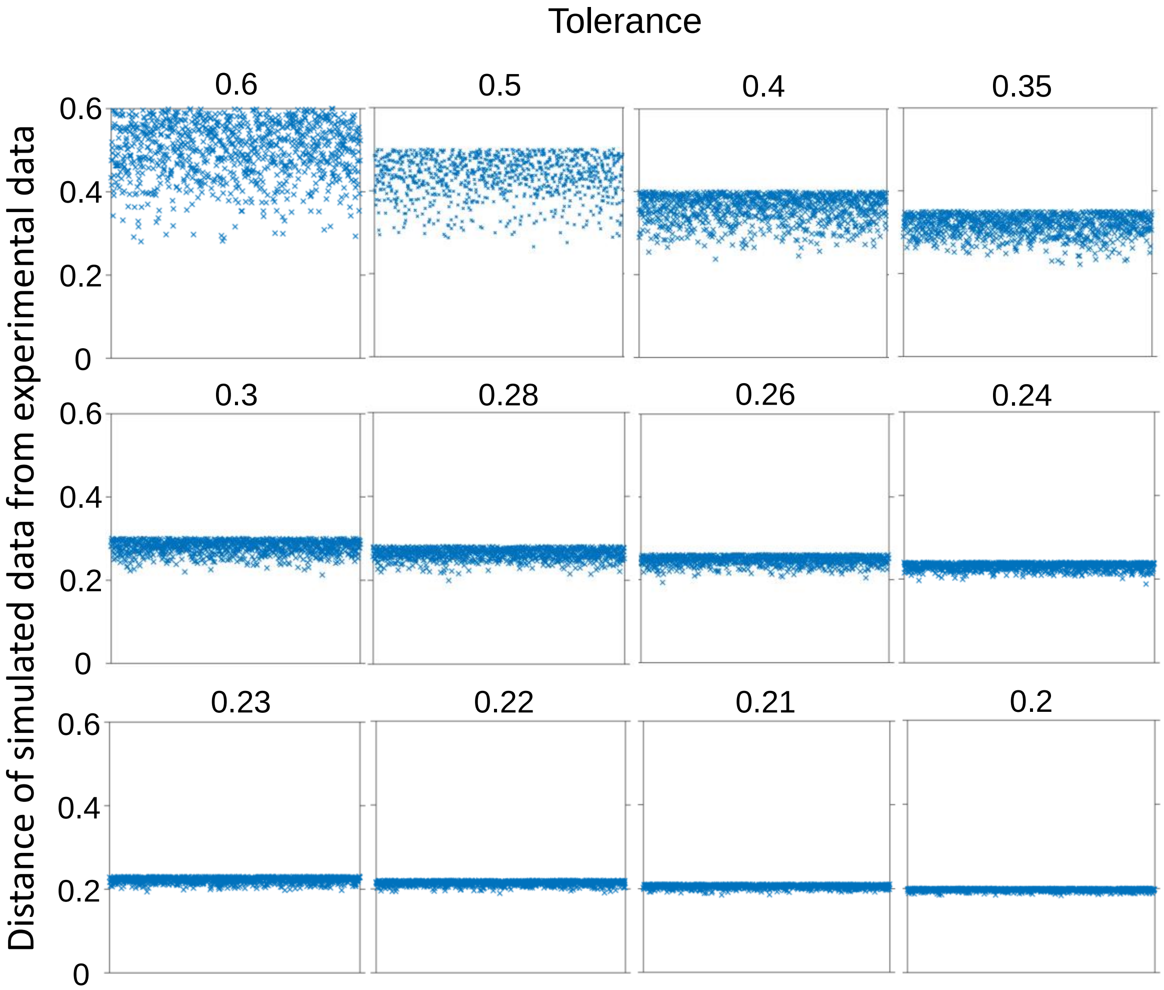

Fig. S3. The fit of the final model improves from generation to generation in the ABC-SMC method. In each generation, the threshold or tolerance for accepting a fit (numbers on top of the panels) was lowered. In the final generation, all accepted fits have a similar distance between simulated and experimental data, showing that fits with further reduced distance did not occur. None of the accepted fits in the first generation would have been accepted in the last generation. The x axis has no meaning and is simply used to spread out the data. See Supplemental Text for the distance measure.

337

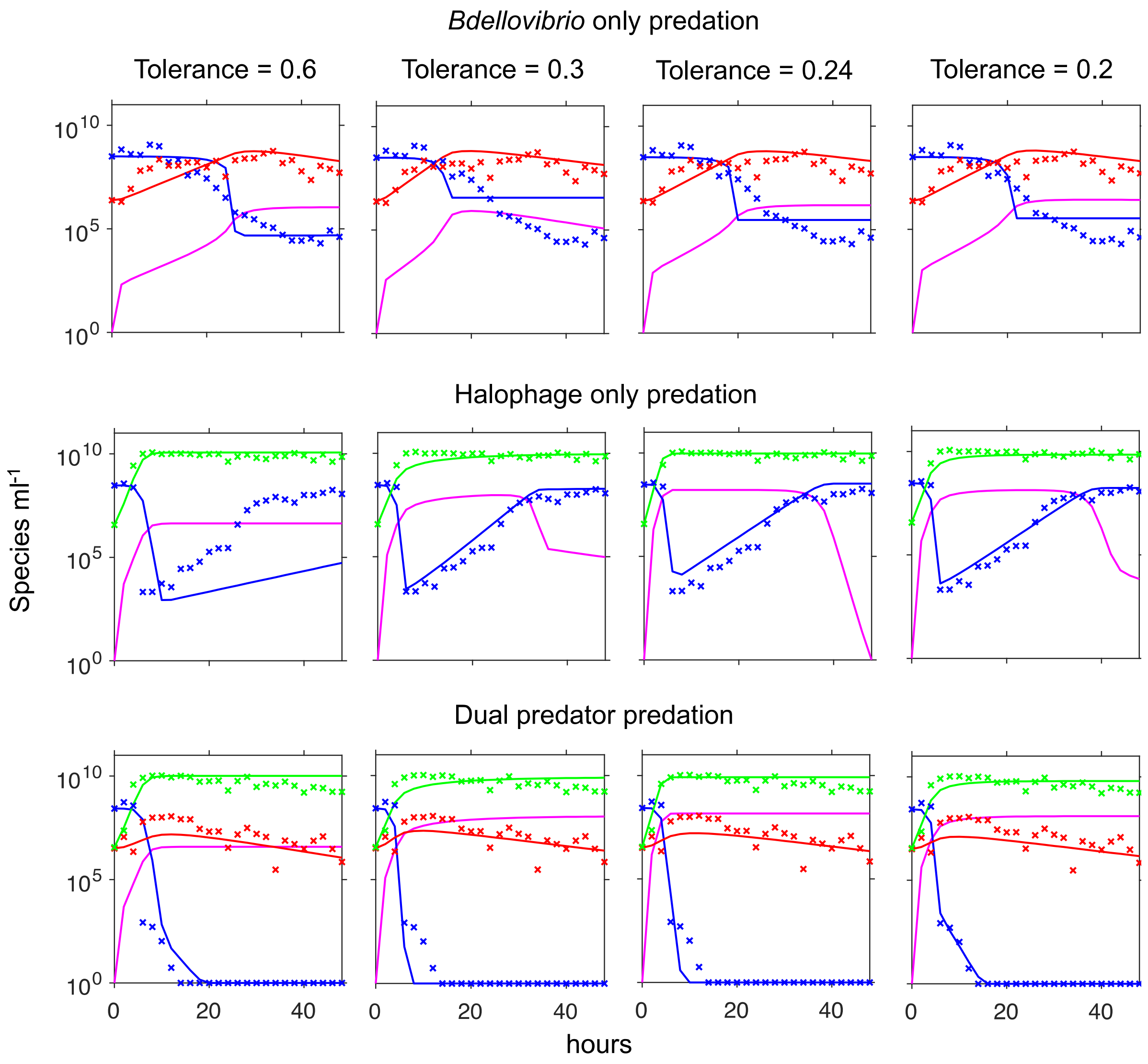

Fig. S4. Model fit improves during ABC-SMC iterations. The fit of the final model to all data improves over the generations, showing a subset of the generations in Fig. S3.

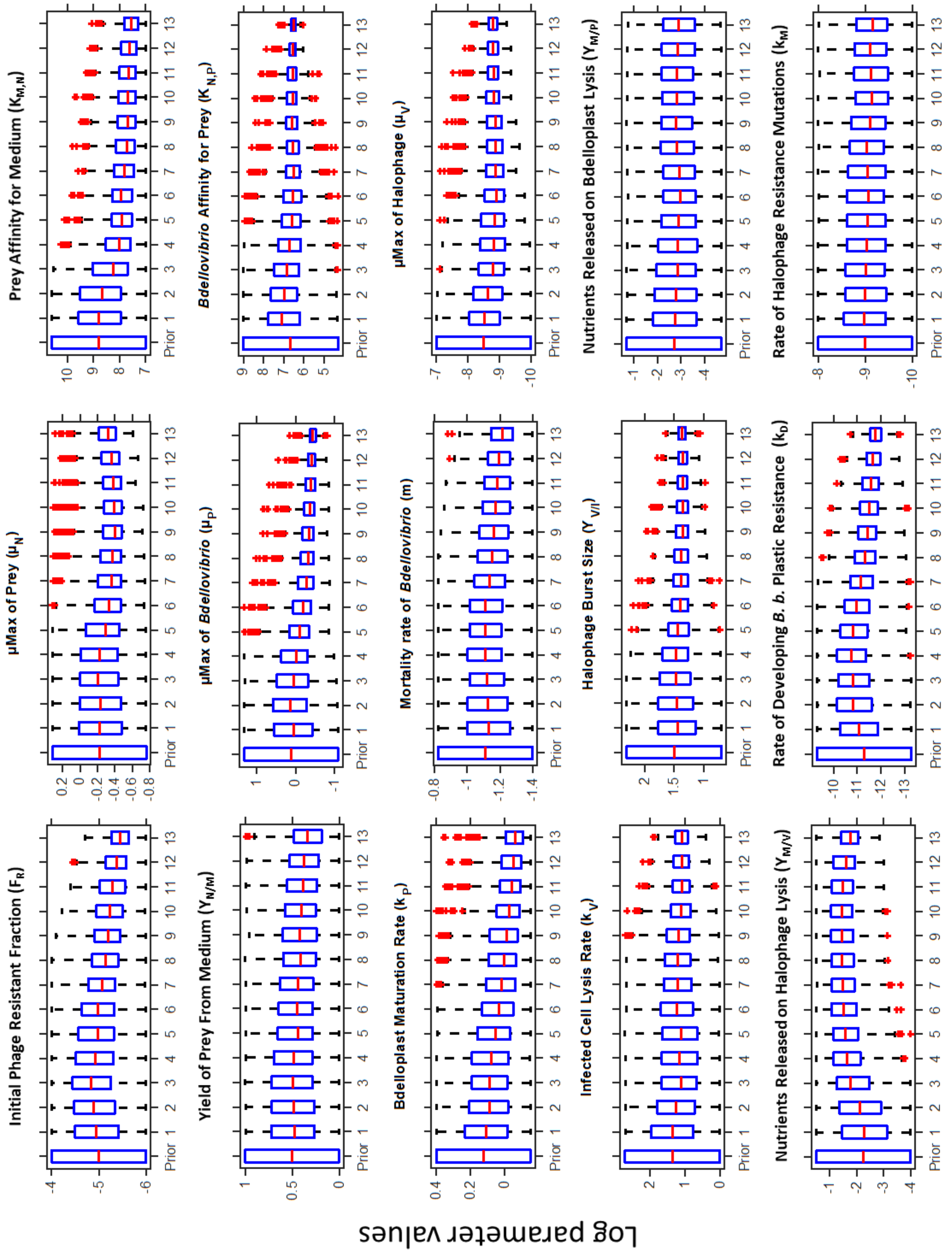

Fig. S5. Convergence of fitted parameters from generation to generation, starting from the uniform priors to the values after the final generation 12. Parameters that are better informed by the experimental data reach a more narrow spread of fitted values. Medians and quartiles from the final generation are shown in Supplementary Table S1. Note the log scale for the parameters.

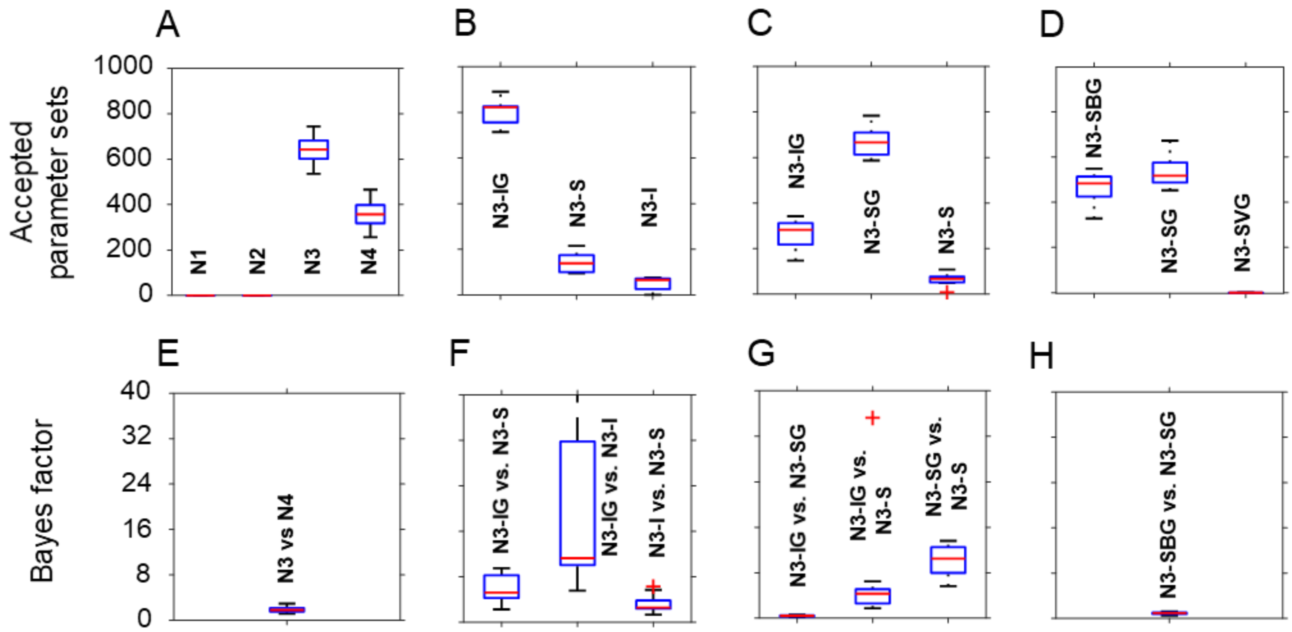

Fig. S6. Box plots showing the variation amongst the 10 repeats of model selection. We repeated the model selection procedure shown in Fig. 4A-D ten times because finding 1,000 acceptable parameter sets with a Monte Carlo method could lead to some variation between runs of the ABC-SMC algorithm and it is good to check whether these differences are large. We only show repeats for sub-model selections where the outcome was not 100% in favour of one model variant. The Bayes factor is the ratio of the times model X was accepted versus model Y.

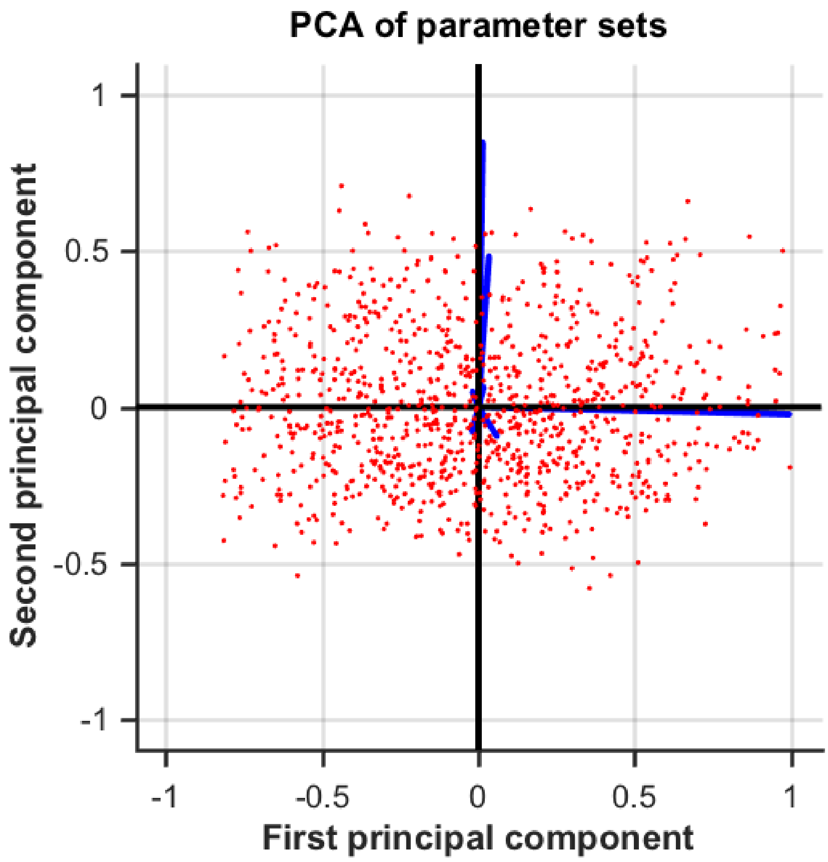

Cumulative % component contributions

1st	44.2
2nd	59.9
3rd	71.0
4th	78.7
5th	85.2
6th	90.7
7th	94.5
8th	96.7
9th	98.5
10th	99.0
11th	99.4
12th	99.7
13th	99.9
14th	100
15th	100

Fig. S7. Objective selection of a typical parameter set. Selection was from the hundreds of parameter sets that gave acceptable fits and were nearly equally good (see Fig. S3). Principal Component Analysis (PCA) was used to find the centre of the cloud of parameter sets in 15 dimensional parameter space. Four parameter sets that were closest to the centre were picked and used to run simulations. Results of these 4 simulations were indistinguishable by eye and one of these 4 parameter sets was then chosen as 'typical' and used in Fig. 5 and reported in Table S1.

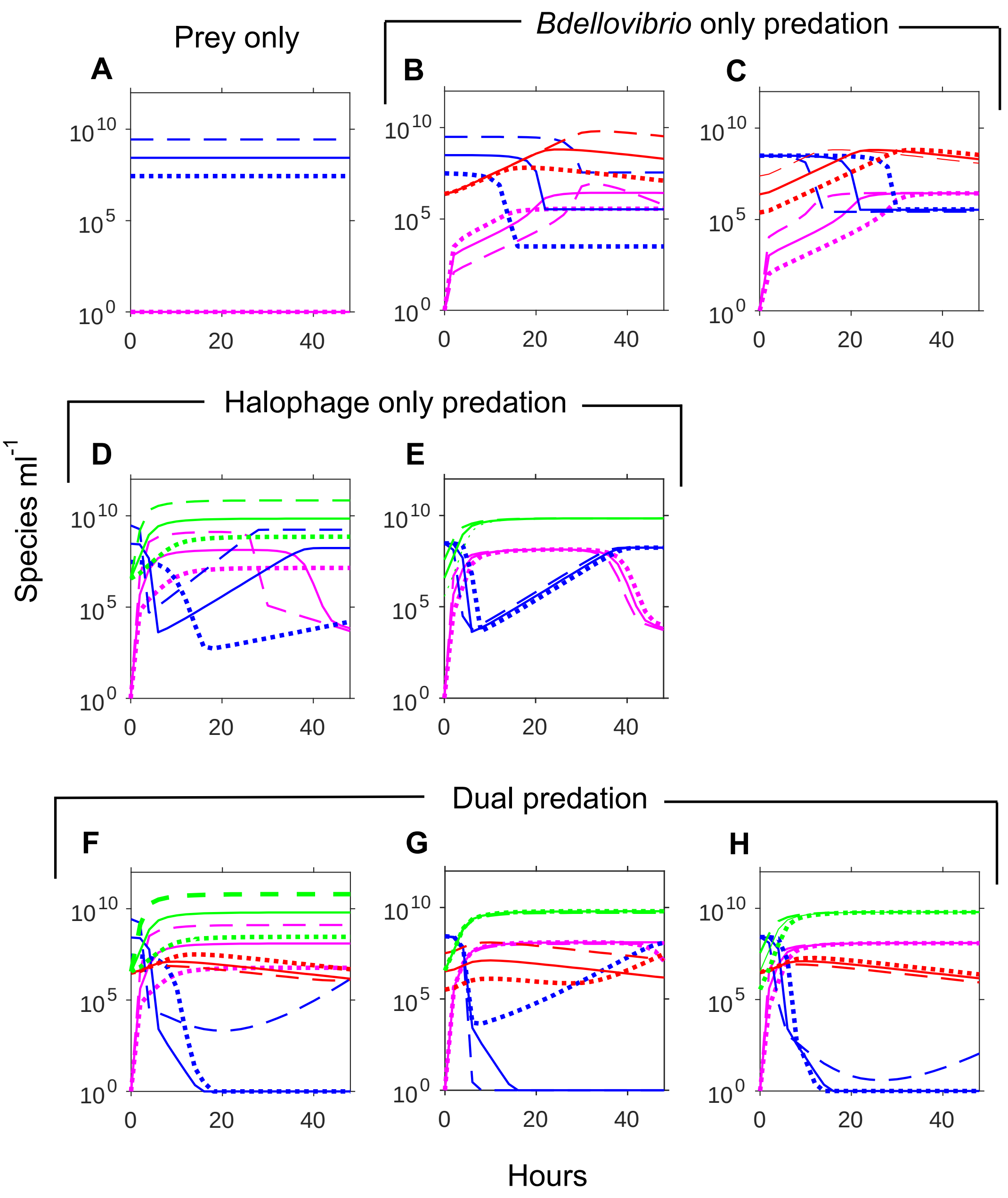

Fig. S8. Effect of varying initial densities. The model was simulated using the typical parameter values fitted to all data in Table S1, with either initial values from the experimental data (solid lines), one order of magnitude higher than these values (dashed lines) or one order of magnitude lower (dotted lines). Blue: *E. coli* prey, Red: *B. bacteriovorus*, Green: bacteriophage halo, Pink: medium. **(A)** Effects of initial prey density on prey only scenario. **(B-C)** Effects of varying either initial prey **(B)** or initial *Bdellovibrio* **(C)** densities on *Bdellovibrio* only predation. **(D-E)** Effects of varying either initial prey **(D)** or initial halophage **(E)** densities on halophage only predation. **(F-H)** Effects of varying either initial prey **(F)**, initial *Bdellovibrio* **(G)** or initial halophage **(H)** densities on dual predation.
